# Molecular phylogeography and species distribution modelling evidence of ‘oceanic’ adaptation for *Actinidia eriantha* with a refugium along the oceanic–continental gradient in a biodiversity hotspot

**DOI:** 10.1186/s12870-022-03464-5

**Published:** 2022-02-28

**Authors:** Rui Guo, Yong-Hua Zhang, Hua-Jie Zhang, Jacob B. Landis, Xu Zhang, Heng-Chang Wang, Xiao-Hong Yao

**Affiliations:** 1 CAS Key Laboratory of Plant Germplasm Enhancement and Specialty Agriculture, Wuhan Botanical Garden, The Chinese Academy of Sciences, Wuhan, 430074 Hubei China; 2 Center of Conservation Biology, Core Botanical Gardens, The Chinese Academy of Sciences, Wuhan, 430074 Hubei China; 3 College of Life Sciences, University of Chinese Academy of Sciences, Beijing, 100049 China; 4 Department of Plant Sciences, University of Cambridge, Tennis Court Road, Cambridge, CB2 3EA UK; 5 College of Life and Environmental Sciences, Wenzhou University, Wenzhou, 325035 China; 6 School of Integrative Plant Science, Section of Plant Biology and the L.H. Bailey Hortorium, Cornell University, Ithaca, NY 14853 USA; 7 BTI Computational Biology Center, Boyce Thompson Institute, Ithaca, NY 14853 USA

**Keywords:** *Actinidia eriantha*, Refugium, Oceanic–continental gradient, ‘Oceanic’ adaptation, Climatic fluctuations, Phylogeography, Subtropical China

## Abstract

**Background:**

Refugia is considered to be critical for maintaining biodiversity; while discerning the type and pattern of refugia is pivotal for our understanding of evolutionary processes in the context of conservation. Interglacial and glacial refugia have been studied throughout subtropical China. However, studies on refugia along the oceanic–continental gradient have largely been ignored. We used a liana *Actinidia eriantha*, which occurs across the eastern moist evergreen broad-leaved forests of subtropical China, as a case study to test hypotheses of refugia along the oceanic–continental gradient and ‘oceanic’ adaptation.

**Results:**

The phylogeographic pattern of *A. eriantha* was explored using a combination of three cpDNA markers and 38 nuclear microsatellite loci, Species distribution modelling and dispersal corridors analysis. Our data showed intermediate levels of genetic diversity [haplotype diversity (*h*_T_) = 0.498; unbiased expected heterozygosity (U*H*_E_) = 0.510] both at the species and population level. Microsatellite loci revealed five clusters largely corresponding to geographic regions. Coalescent time of cpDNA lineages was dated to the middle Pliocene (ca. 4.03 Ma). Both geographic distance and climate difference have important roles for intraspecific divergence of the species. The Zhejiang-Fujian Hilly Region was demonstrated to be a refugium along the oceanic–continental gradient of the species and fit the ‘refugia in refugia’ pattern. Species distribution modelling analysis indicated that Precipitation of Coldest Quarter (importance of 44%), Temperature Seasonality (29%) and Mean Temperature of Wettest Quarter (25%) contributed the most to model development. By checking the isolines in the three climate layers, we found that *A. eriantha* prefer higher precipitation during the coldest quarter, lower seasonal temperature difference and lower mean temperature during the wettest quarter, which correspond to ‘oceanic’ adaptation. *Actinidia eriantha* expanded to its western distribution range along the dispersal corridor repeatedly during the glacial periods.

**Conclusions:**

Overall, our results provide integrated evidence demonstrating that the Zhejiang-Fujian Hilly Region is a refugium along the oceanic–continental gradient of *Actinidia eriantha* in subtropical China and that speciation is attributed to ‘oceanic’ adaptation. This study gives a deeper understanding of the refugia in subtropical China and will contribute to the conservation and utilization of kiwifruit wild resources in the context of climate change.

**Supplementary Information:**

The online version contains supplementary material available at 10.1186/s12870-022-03464-5.

## Background

Distinguishing the type of refugia is crucial for our understanding of evolutionary processes such as adaptation and speciation, while also forecasting how current climate change may affect the species in the context of conservation [1, 2]. Refugia are habitats for which components of biodiversity retreat to, persist in and can potentially expand from under changing environmental conditions [2]. Refugia are mainly classified into interglacial refugia, glacial refugia and refugia along the oceanic–continental gradient [1]. Interglacial and glacial refugia are usually defined within a latitudinal gradient, while refugia along the oceanic–continental gradient often have a longitudinal perspective [1]. The oceanic-continental gradient in climate provides increasing seasonal range of temperature and decreasing precipitation from the coast to inland areas [3]. Mountain ranges, especially near the coast, intercept clouds and cause major local increase in precipitation [3]. Two types of adaptation have been defined for species with refugia along the oceanic–continental gradient: ‘oceanic’ and ‘continental’. ‘Oceanic’ adaptation implies more humid, less seasonably variable climate; ‘continental’ adaptation consists of a drier climate with greater seasonal variation [1]. Molecular phylogeographic studies at the intraspecific level can reveal patterns of historical demography of species, including areas of refugia [4].

Extensive literature has recently emerged on the phylogeographic patterns of plant species in subtropical China (e.g. [5–10]). Subtropical China is a global biodiversity hotspot, well known due to its species richness, complex topography and fluctuating paleoclimate [11–15]. This area is located between the Qinling Mountains-Huai River line (ca. 34°N) and the Tropic of Cancer (ca. 23°N), bordered by the Qinghai-Tibetan Plateau (ca. 99°E) in the west and the coastline in the east [16]. This region has never been directly covered by ice sheets during glaciation periods of the Pleistocene, thus has preserved numerous Tertiary plant genera [16, 17]. However, the area has undergone complex climate changes during the Quaternary Period. For example, the climate of this region during the LGM (last glacial maxima) was cooler by c. 4–6 °C and dryer by c. 400–600 mm/yr [18, 19]. Interglacial and glacial refugia have been studied thoroughly in subtropical China, shedding light on our understanding of the effects of glacial and postglacial cycles. For example, glacial refugia were detected for *Lindera aggregata* (Sims) Kosterm [9], *Castonopsis eyrei* (Champ.) Tutch [20]., *Machilus thunbergii* Sieb. et Zucc [21]., *Loropetalum chinensis* (R. Br.) Oliver [22] and *Sargentodoxa cuneata* (Oliv.) Rehd. et Wils [23] while interglacial refugia were identified for *Emmenopterys henry* Oliv [12]. and *Rosa sericea* Lindl. complex [24]. Large mountains in subtropical China served as important refugia such as the Nanling Mountains for *Lindera aggregata* [9]. The variable topography in these areas offer a large scope of elevational shifts for plant species in response to climatic changes [2]. Despite the gradient of oceanic–continental climate bing significantly variable during glacial cycles with important biotic consequences [25], refugia along the oceanic–continental gradient were largely ignored in the area.

*Actinidia eriantha* Benth. (Ericales: Actinidiaceae; 2n = 58) is a suitable model for testing hypotheses of refugia along the oceanic–continental gradient and ‘oceanic’ adaptation, on account of its distribution and moist habitat. The species is an important component of liana species in eastern moist evergreen broad-leaved forests (EBLF) of subtropical China at an altitude of 200 to 1500 m [26]. The more narrow distribution of the species along a series of mountains from the coastline of the East China Sea to the eastern edge of the Yungui Plateau implies an oceanic adaptation. *Actinidia eriantha* is a functionally dioecious, perennial liana species characterized by young branchlets, petioles, inflorescences, sepals and fruits densely tomentose with milky-white to dirty yellow hairs or appressed tomenta [27]. The species is recognized as a valuable species for commercial kiwifruit improvement by extending shelf life and increasing vitamin C concentration, as well as having been used in traditional Chinese medicine to treat gastric carcinoma, nasopharyngeal carcinoma, breast carcinoma, and hepatitis [28]. To assist in collecting germplasm resources and protection of the species, revealing how the species responded to past climate changes and where refugia are located is necessary. To date, a few studies of population genetic differentiation of *A. eriantha* with limited sampling from narrow geographical areas have been conducted [29–31]. The lack of collections in many areas of the region has prevented the phylogeographic studies for conservation purposes.

Here, we used extensive sampling covering the whole distribution range of the species combined with nuclear SSR and cpDNA evidence, Species Distribution Models (SDM) and dispersal corridors analyses to explore the patterns of historical demography of *A. eriantha* comprehensively. We asked: i) What are the patterns of genetic diversity and population divergence of *A. eriantha*? ii) Which factors have an effect on the genetic structure of the species? iii) What is the location and type of the refugia of the species? iv) What is the direction of dispersal of the species? Our goals are hence to test the hypotheses of refugia along the oceanic–continental gradient and ‘oceanic’ adaptation, revealing how the species responded to past climate changes and provide information for collecting kiwifruit germplasm resources and conserve the species.

## Results

### Chloroplast DNA haplotype diversity

Three noncoding cpDNA regions were concatenated for individuals of *A. eriantha* and two outgroup species i.e. *A. fulvicoma* Hance and *A. chinensis* Planch., with a complete length of 1605 bp. Twenty-three cpDNA haplotypes were identified within *A. eriantha*, with eight being singletons (Additional file 1), including 16 substitutions and nine indels (1-19 bp). Our data showed intermediate levels of haplotype diversity (*h*_T_ = 0.498) and nucleotide diversity (π_T_ = 9.1 × 10^− 4^) within the species. Significant phylogeographic structure (*N*_ST_ = 0.592 > *G*_ST_ = 0.447, *P* < 0.001) was detected. In the SAMOVA analyses, *K* = 3 when *F*_CT_ values reaches the maximum (0.821), which means that three regional groups of populations were identified. These three groups are denoted as “Southeast edge”, “Southwest edge” and “Main part” according to their relative positions in the distribution area of the species. The “Southeast edge” and “Southwest edge” groups only contain a single population, i.e. HA in “Southeast edge” and LP in “Southwest edge”. Hierarchical AMOVA showed a great amount of variation (82.1%) occurred among the three regional population groups and only 6.6% presented differences among populations within groups, and 11.3% of the variation within populations (Additional file 2).

**Fig. 1:**
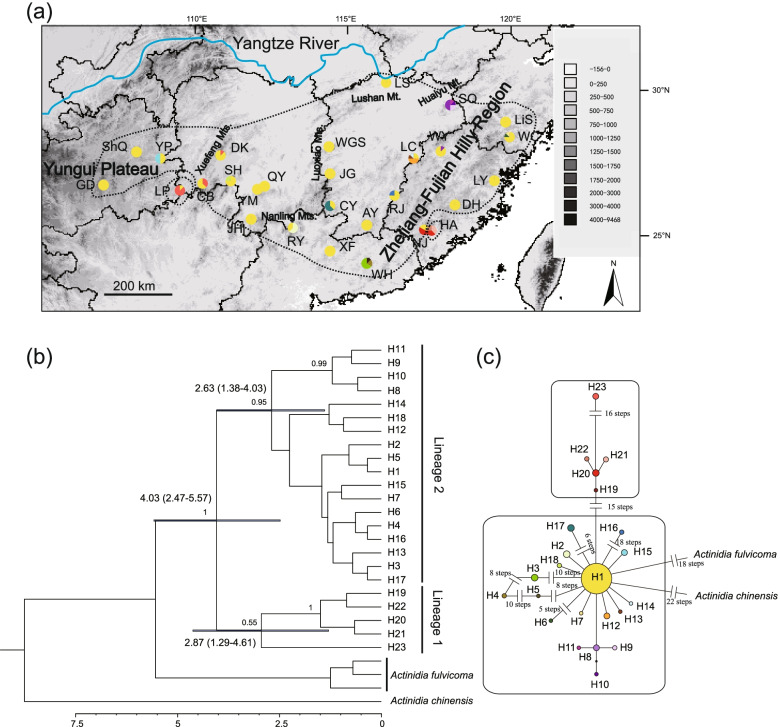
Geographical distribution of *A. eriantha* cpDNA haplotypes, BEAST-derived chronograms and TCS network. **a** Geographical locations of the 28 populations and distributions of 23 chloroplast haplotypes of *A. eriantha* examined in this study (the scale on the map represents meters above sea level). The three dashed lines correspond to the three population groups (“Southeast”, “Southwest” and “Main part”) identified by the program SAMOVA. **b** BEAST-derived chronogram of *A. eriantha* based on cpDNA sequences. Blue bars indicate 95% HPD credibility intervals for nodes of particular interest with ages (in Myr ago, Ma). Only bootstrap values higher than 50% are denoted above branches. **c** TCS-derived network of 23 chloroplast haplotypes. Each circle means a unique haplotype, with circle size reflecting its frequency. Small black circles mean missing haplotypes

The network of 23 haplotypes displayed a star-like pattern, with eight haplotypes directly connected to H1 by one mutation (Fig. 1c). H1 was designated as an ancestral haplotype since it was most closely related to the outgroup and occupied an interior position in the network. The dominant haplotype H1 occurred in 156 individuals (70.6%) from 25 populations except for SQ, WH and HA, three populations located in the Zhejiang-Fujian Hilly Region (Fig. 1a). However, most haplotypes (19/23) were restricted to a single population (Additional file 1). Two haplotype lineages were recognized based on the results of network analysis and Bayesian phylogenetic inference (Fig. 1b and c). The split between Lineage 1 and Lineage 2 was dated to 4.03 Ma (95% HPD: 2.47-5.57). Haplotype lineages showed distinct distribution ranges. The haplotypes in Lineage 2 (H8, H9, H10 and H11) were found exclusively in the Northwest part of the Zhejiang-Fujian Hilly Region. In Lineage 1, H20, H21 and H22 appeared only in the southern portion of the Zhejiang-Fujian Hilly Region, while H23 occurs in the Xuefeng Mountains.

**Fig. 2:**
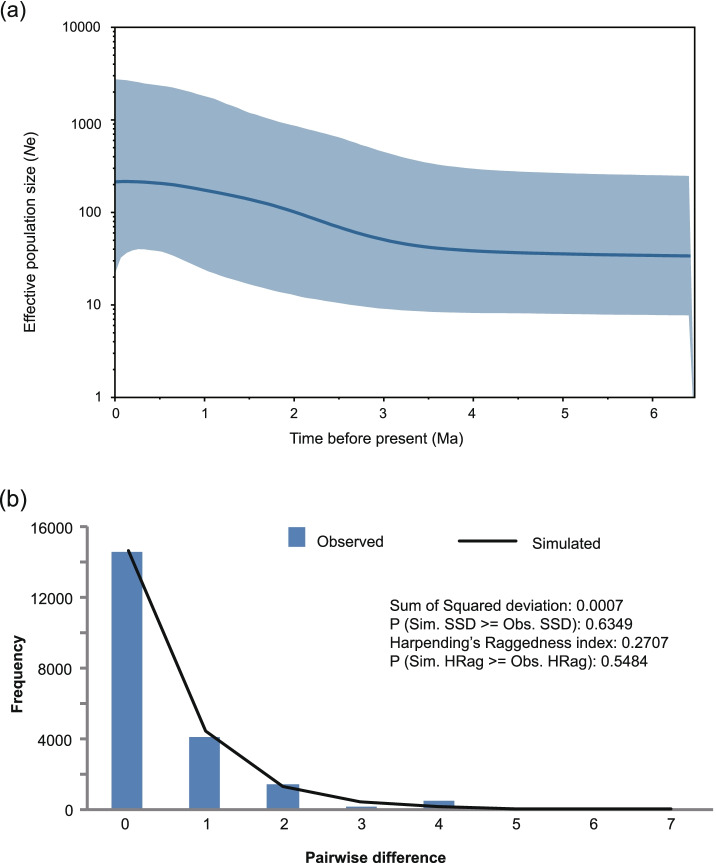
The results of bayesian skyline plots (BSP) and mismatch distribution analysis (MDA) of the “Main part” inferred from *A. eriantha* cpDNA. **a** Bayesian skyline plots (BSP) estimated using BEAST2 v. 2.4. The thick solid blue line is the median estimate, and the area delimited by the light blue broadband represents the highest posterior density (HPD) 95% confidence intervals for *N*e. **b** Mismatch distribution analysis (MDA) estimated in Arlequin v. 3.5

The regional population group “Main part”, which contains all haplotypes in Lineage 2, was used for mismatch distribution analysis (MDA) and bayesian skyline plots (BSP) to estimate the possible population spatial expansion indicated by the clear star-like phylogeny of haplotypes in Lineage 2. The BSP showed a slight population expansion occurring between c. 3.5 and 1.5 Ma (Fig. 2a). However, this analysis cannot precisely estimate *N*_e_ because of the very broad confidence intervals. The result of the MDA showed that the observed distribution of pairwise differences among the haplotypes do not differ significantly from the expected distribution under the sudden-expansion model (SSD = 0.0007, *P* = 0.6349; HRag = 0.2707, *P* = 0.5484) (Fig. 2b). The time of spatial sudden expansion was estimated at 81,775 yr BP (95% CI: 10,112-95,404) based on the parameter τ (3.00; 95% CI: 0.371-3.5). The significantly negative values of Tajima’s *D* (− 2.06, *P* < 0.05) and Fu’s *F*_S_ (− 16.32, *P* < 0.05) also supported that the “Main part” had experienced regional expansions in the past. Since similar results were estimated for all populations of the species, we did not show them here. The small size and low number of haplotypes of the other regional groups “Southeast edge” and “Southwest edge” are unsuitable for estimating possible population spatial expansion, therefore no MDA, BSP and neutrality tests were conducted for them.

Six dispersal and six vicariance events were discovered by BBM (Bayesian Binary MCMC) analysis. The ‘east area’ was identified as the most likely ancestral area of the *A. eriantha* (Additional file 3).

### Nuclear microsatellite loci data analysis

Among the 38 loci, two were detected as outliers by Arlequin (AET22 and AET167) and six by SamBada (UDK96-040, AET38, AET76, AET97, AET104 and AET167). Of these, locus AET167 was detected as an outlier by both tests and considered a locus under selection. The locus is significantly correlated with four bioclimatic variables: Temperature Seasonality (BIO4), Annual Precipitation (BIO12), Precipitation of Driest Month (BIO14) and Precipitation of Coldest Quarter (BIO19). No genetic function was annotated for the sequence containing AET167 by GO analyses. In total, 31 loci (including 26 EST-SSRs and 5 genomic SSRs) displayed no evidence of selection in either outlier tests were therefore considered neutral loci.

We detected a relatively high total number of alleles per locus (mean 12.7, range 4-32). For all 38 loci, *H*_E_ ranged from 0.293 to 0.938. Over all populations, 22 of the 38 loci displayed a significant heterozygosity deficit (Additional file 4). Significant deviations from Hardy-Weinberg equilibrium were detected in 86 of 1064 population-locus comparisons, yet no consistent patterns across loci or populations were evident. Null alleles were detected in 102 of 1064 combinations, which was above the expected number (53) by chance at the 5% level, albeit with no significant pattern specific to a population or locus. Linkage disequilibrium indicated significant deviations for 62 of 19,684 population-locus-locus combinations, which were lower than the expected number (984) by chance at the 5% level, suggesting no significant Linkage Disequilibrium among the 38 loci.

**Fig. 3:**
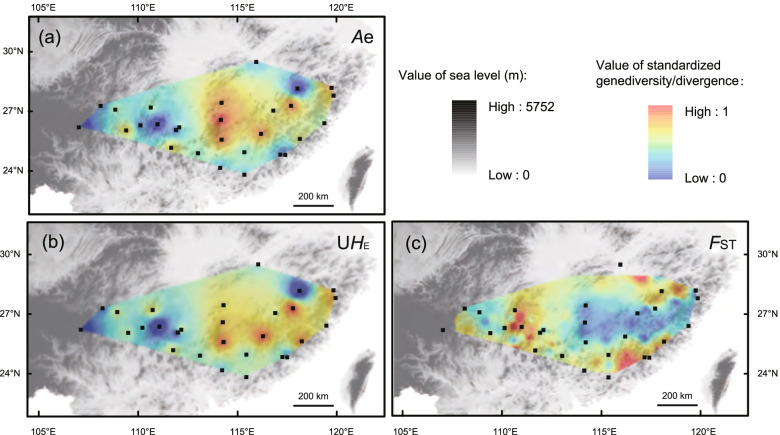
Genetic landscapes for *A. eriantha*: **a** genetic diversity based on *A*e (No. of effective alleles); **b** genetic diversity based on *UH*_e_ (unbiased expected heterozygosity); **c** genetic divergence based on *F*_ST_ [*F*_ST_ = (*H*_t_ - *H*_e_) / *H*_t_, *H*_t_ means total expected heterozygosity, *H*_e_ means expected Heterozygosity]. The values of *A*_e_, *UH*_e_, *F*_ST_ have been standardized as [0,1]

For each population across the 31 neutral loci, the genetic diversity parameters are listed in detail (Additional file 5). Values for *Ae* (mean 2.52, range 1.66-3.11), *R*_S_ (mean 2.781, range 2.014-3.240)*, H*_o_ (mean 0.452, range 0.326-0.542) and U*H*_E_ (mean 0.510, range 0.324-0.614) indicate moderate diversity within populations. Significant heterozygosity deficit was displayed in 21 of the 28 populations. Higher diversity was identified in eastern *A. eriantha* populations (except for several marginal populations) (Fig. 3ab). Private alleles (*A*p) occurred in all populations except YM and DK.

**Fig. 4:**
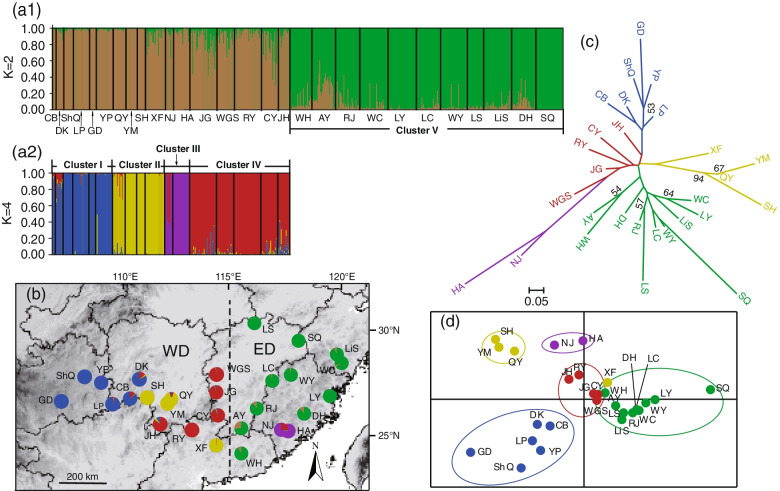
Population genetic structure of *Actinidia eriantha.***a** Histogram of the Bayesian assignment for 28 populations (629 individuals) and the hierarchical Bayesian assignment for 17 populations (293 individuals) of *A. eriantha* based on genetic variation at 31 neutral EST-SSR loci using STRUCTURE. Each vertical bar represents one individual and its probability of membership for each of the *K* = 2 and hierarchical K = 4 clusters. **b** Geographic origin of the 28 *A. eriantha* populations and their color-coded grouping according to the STRUCTURE analysis. **c** The un-rooted NJ tree of 28 population revealed by 31 neutral nSSR data. **d** Principal coordinates analysis (PCoA) of *A. eriantha* based on their genetic distances (*D*_A_) derived from 31 neutral nSSRs

The genetic divergence pattern showed that eastern *A. eriantha* populations (except for several marginal populations) possessed lower values of genetic divergence than western populations (Fig. 3c). The Bayesian assignment indicated *K* = 2 was the best when all populations were included (Additional file 6). Eastern populations (except for HA and NJ) were grouped into one cluster (Cluster V), and the rest were grouped into another cluster (Fig. 4a1). Subsequent hierarchical analyses split the rest of the species into four clusters: Cluster I, II, II and IV (Additional file 6 and Fig. 4a2). Overall, five clusters were identified, which were roughly consistent with the east-west distribution of the species (Fig. 4b). The PCoA and the unrooted NJ tree indicated a distinct differentiation among populations (Fig. 4c and d), in line with the findings of Bayesian assignment.

Historical gene flow among clusters (*m*_h_) range from 0.001 (0-0.006) to 0.169 (0.155-0.173) (Additional file 7). Higher gene flow was estimated from cluster V to cluster I (0.082) and cluster V to cluster IV (0.169) unidirectionally. BayesAss yielded lower contemporary gene flow among most clusters than historical gene flow, with *m*_c_ ranging from 0.001 (0-0.003) to 0.047 (0.012-0.085) (Additional file 7).

**Fig. 5:**
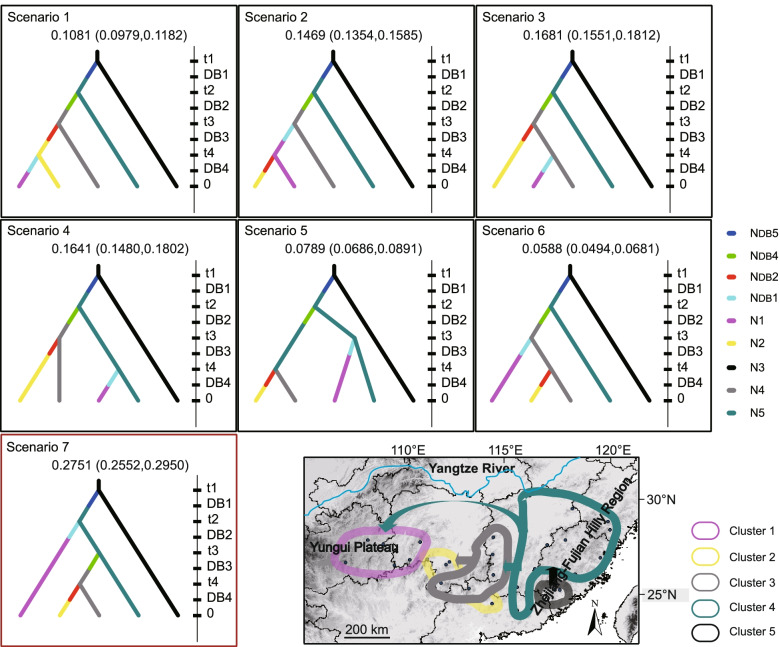
Seven evolutionary scenarios for five clusters of *A. eriantha* tested with approximate Bayesian computation (ABC) analyses using DIYABC. Prior distributions of model parameters were set for effective population size of five sampled clusters (N1–N5) and 4 founder clusters (N1F–N5F, except for N3F), duration of bottleneck after colonization event (DB) and relative timing of events in number of generations (t1–t4). Posterior probabilities of the seven scenarios obtained by logistic regression of 1% of the closest simulated datasets are shown on the top of each scenario. Scenario outlined in red is the best option

Scenarios of historic processes were evaluated in a three-step analysis (see Additional file 8 for prior distributions). For the first set of analyses aiming to evaluate the relationship among the source genetic units (set A), the relative posterior probabilities calculated for each scenario provided the strongest statistical support for scenario A2 (0.5738, 95% CI: 0.5567-0.5908) (Additional file 9), suggesting that Cluster V diverged from Cluster III. Second, based on the results from set A, three sets of scenarios were designed to analyze which source genetic unit Cluster I, II or IV diverged from (set B, C and D). The relative posterior probabilities calculated for each scenario of simulation set B, C and D, provided the strongest statistical support for Set B1 (0.6098, 95% CI: 0.5970-0.6227), C1 (0.5839, 95% CI: 0.5750-0.5927) and D1 (0.6344, 95% CI: 0.6156-0.6533) (Additional file 9), suggesting a common eastern origin (Cluster V) for the three colonization genetic units. Finally, Set E was calculated to test the relationship among colonization genetic units, based on the results from set B, C and D. Set E7 (0.2751, 95% CI: 0.2552-0.2950) was identified as the best-fit scenario (Fig. 5), indicated that Cluster I and Cluster IV diverged from Cluster V in chronological order, followed by the divergence of Cluster II from Cluster IV. We also estimated the divergence time and the population sizes for the five clusters (Additional file 10). The estimates in Set E were used since they are more credible when all populations were included in the set while the estimates from other sets may be biased due to missing segments of the populations. The time parameters are converted into years by multiplying generation time, which was set to 7 years for *A. eriantha*. Cluster V diverged from the ancestral population of *A. eriantha* Cluster III at approximately 460,000 generations ago (95% CI: 105,000 - 2,650,000) which corresponds to c. 3.22 Ma (95% CI: 0.74 - 18.55). Then, Cluster I and Cluster IV diverged from Cluster V at approximately 294,000 (95% CI: 87,900 - 1,290,000) and 321,000 (95% CI: 107,000 - 785,000) generations ago, or c. 2.06 Ma (95% CI: 0.62 - 9.03) and 2.25 Ma (95% CI: 0.75 - 5.50), respectively. The split between Cluster II and Cluster IV was estimated to approximately 143,000 generations ago (95% CI: 38,000 - 360,000) or c. 1.00 Ma (95% CI: 0.27 - 2.52). No bottleneck was found by comparing the population sizes of Ni and these of N_DB_i (i = 1, 2, 4). The population size of the species expended along with divergence from 522,000 (N3) to 3,145,000 (N1 + N2 + N3 + N4 + N5).

### IBD and IBE analyses

The *R*^*2*^ value for multiple matrix regression with randomization (MMRR) simulation based on the SSR neutral data was moderate (*R*^*2*^ = 0.127, *P* = 0.002), suggesting a significant fit to this data, albeit not for cpDNA (*R*^*2*^ = 0.001, *P* = 0.968). The MMRR results (*β*_*D*_ = 0.279, *P* < 0.001; *β*_*E*_ = 0.139, *P* = 0.228) indicated that geographic distance had a significant effect on neutral SSR divergence, whereas the effects of environment factors were not significant (Table 1). For cpDNA divergence, there was no evidence of IBD or IBE (*β*_*D*_ = 0.017, *P* = 0.823; *β*_*E*_ = 0.014, *P* = 0.933; Table 1). The partial Mantel test also revealed similar results (SSR: *r*_*D*_ = 0.267, *P* < 0.001; *r*_*E*_ = 0.137, *P* = 0.228; cpDNA: *r*_*D*_ = 0.016, *P* = 0.364; *r*_*E*_ = 0.013, *P* = 0.470, Table 1).

**Table 1:** Results of Multiple Matrix Regression with Randomization (MMRR) analysis and partial Mantel test for SSR and cpDNA dataset of *Actinidia eriantha*

	MMRR						Partial Mantel test			
	Model		IBD		IBE		IBD		IBE	
	*R*^*2*^	*P*	*β*_*D*_	*P*	*β*_*E*_	*P*	*r*_*D*_	*P*	*r*_*E*_	*P*
SSR	**0.127**	0.002	**0.279**	< 0.001	0.139	0.228	**0.267**	< 0.001	0.137	0.127
cpDNA	0.001	0.968	0.017	0.823	0.014	0.933	0.016	0.364	0.013	0.470

*IBD* Isolation by distance, *IBE* Isolation by environment

### Species distribution models and dispersal corridors

**Fig. 6:**
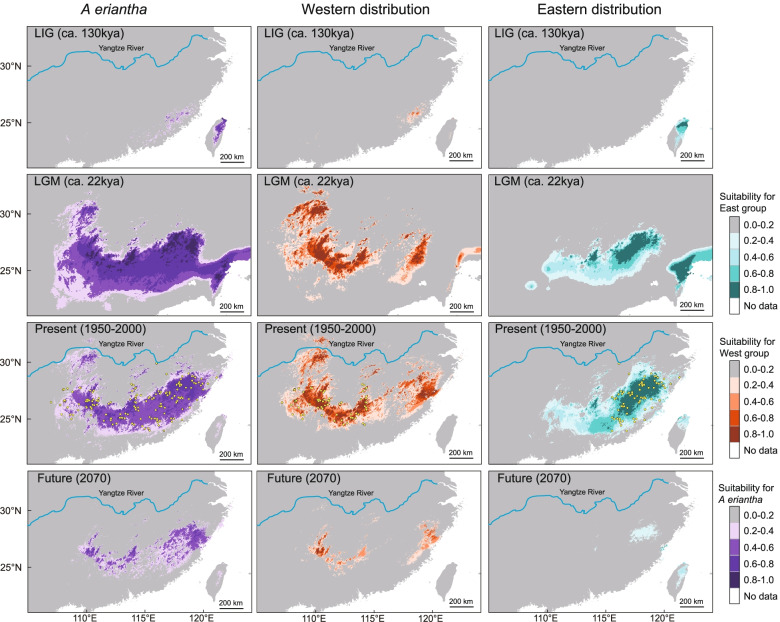
Potential distributions of *A. eriantha*, West Distribution (WD) and East Distribution (ED) predicted using MaxEnt based on five bioclimatic variables representing the LIG, LGM, current and future climatic conditions, respectively. Warmer colors denote areas with a higher probability of presence. Dots show the extant occurrence points of the *A. eriantha*

The Maxent models for *A. eriantha* had high predictive power and did not over-fit the present data (AUC values = 0.909). Our analysis indicated that Precipitation of Coldest Quarter (BIO19; importance of 44%), Temperature Seasonality (BIO4; 29%) and Mean Temperature of Wettest Quarter (BIO8; 25%) contributed the most to model development. The ensemble model for each period is illustrated in Fig. 6. The current potential distribution areas (defined as modelled suitability ≥0.6) of *A. eriantha* generally matches observed distributions, except for predicted but unsupported areas north of the Yangtze River. During the LIG, *A. eriantha* experienced a drastic contraction compared to its current range with scattered areas in the Zhejiang-Fujian Hilly Region near the East China Sea and the eastern part of Taiwan province. Under LGM conditions, the estimated climatic suitability for *A. eriantha* underwent a radical range expansion with continuous suitable habitat from the Yungui Plateau to Taiwan. The future potential areas are contracted, fragmenting into several small suitable habitats scattered in the current distribution range of the species. The Zhejiang-Fujian Hilly Region (the current distribution area of Cluster V and Cluster III) near the East China Sea was always included in the predicted potential distribution areas of the species during the four glacial/interglacial periods (Fig. 6). By comparing the isolines in the three climate layers during the four glacial/interglacial periods, we found that the expansion of *A. eriantha* was accompanied by higher precipitation during the coldest quarter, lower seasonal temperature difference and lower mean temperature during the wettest quarter throughout the distribution area (Additional file 11).

According to the western distribution (WD) and the eastern distribution (ED) range of the species, 158 occurrence points of *A. eriantha* were divided into ED (13 sampled locations and 63 herbaria specimens) and WD (15 sampled locations, 61 herbaria specimens and six location studied by Liu et al. [29] (see Additional file 12 for details). An additional 10 occurrences were not assigned into either group since they are located on the geographic boundary of the two distribution ranges. The Maxent models for both ED and WD had high predictive power and did not over-fit the present data (AUC values = 0.942 and 0.936, respectively). Precipitation of Coldest Quarter (BIO19; importance of 64.8%) and Temperature Seasonality (BIO4; 24.6%) contributed the most to model development for ED, while Temperature Seasonality (BIO4; 31.8%), Precipitation of Coldest Quarter (BIO19; 28.6%) and Mean Temperature of Wettest Quarter (BIO8; importance of 22.7%) contributed the most to model development for WD. The current potential distribution areas of WD not only match observed distributions, but also encompass part of the observed distribution of the ED and unsupported areas north of the Yangtze River. The current potential distribution areas of ED generally match observed distributions. Both WD and ED experienced a drastic contraction during the LIG, a radical range expansion during the LGM and a drastic contraction in the Future (2070). However, WD contracted into the Zhejiang-Fujian Hilly Region while ED contracted into Taiwan during the LIG. Under LGM conditions, potential distribution areas of both parts are similar to current ones, except for the areas of expansion to the northern part of Taiwan province. The future potential areas of WD are contracted to several small suitable habitats scattered in the current distribution range of the species, while the future potential areas of ED are contracted to several peninsulas and islands in Fujian province next to the East China Sea. Observed values of Schoener’s *D* and Warren et al.’s *I* (0.44 and 0.72, respectively) were both significantly lower (*P* < 0.01) than expected from a random distribution (Additional file 13), suggesting the existence of niche differentiation between WD and ED.

**Fig. 7:**
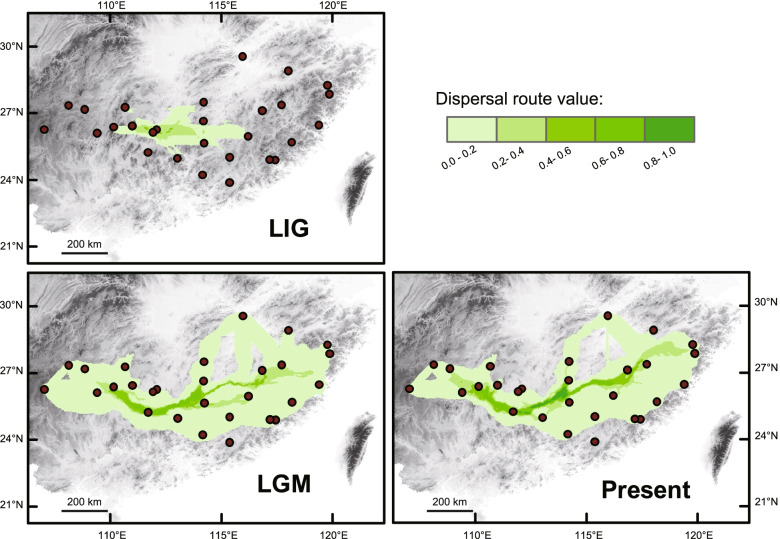
The dispersal corridors for *A. eriantha* based on chloroplast haplotypes at the LIG, LGM and current, respectively. The values of dispersal route have been standardized as [0,1]

The dispersal corridor result revealed a continuously east-west route during the LGM and the present but was hardly present during the LIG (Fig. 7).

## Discussion

### Genetic diversity and genetic structure of *Actinidia eriantha*

Patterns of genetic diversity of *A. eriantha* were investigated with neutral nuclear SSR loci and cpDNA data. At both the species and population levels, our results reveal a moderate level of microsatellite genetic diversity (mean U*H*_E_ = 0.510) and haplotype diversity (*h*_T_ = 0.498) across the 28 populations. The lower values of *H*_o_ than that of U*H*_E_ in most populations possibly resulted from inbreeding, which was confirmed by the significant heterozygosity deficit displayed in 21 of the 28 populations (Additional file 5). Genetic diversity values within populations of *A. eriantha* were slightly lower than that of other plants with a similar habitat, life history and breeding strategies [32]. However, genetic diversity at the population level of *A. eriantha* is likely underestimated. To our knowledge, EST-SSRs often display lower polymorphism than that of genomic SSRs [33]. In fact, genomic SSRs showed higher levels of genetic diversity than EST-SSRs in *A. eriantha* (Additional file 4) and 26 EST-SSRs were included to assess the genetic diversity of *A. eriantha* in the present study. The high genetic diversity at the population level (mean *H*_E_ = 0.763) was previously observed in six populations located at the border between Hunan and Guangxi Province (nine genomic microsatellite loci) [29]. The high level of genetic diversity within populations for the predominantly insect-pollinated species is most likely explained by the outcrossing breeding system, high longevity and high inherent variability of the ancestral species of *A. eriantha*.

The genetic differentiation based on SSR markers (*F*_ST_ = 0.177) are almost identical to the mean values of genetic differentiation among populations of the late-successional (*F*_ST_ = 0.17) or long-lived (*F*_ST_ = 0.19) plants [32] and consistent with previous findings (*F*_ST_ = 0.116) [29]. The low levels of genetic differentiation may result from high levels of historical gene flow among the studied populations (Additional file 7). The significant correlation between genetic and geographic distances (Additional file 7) implies that gene flow is important in shaping the genetic differentiation. The fruit of *A. eriantha* is a relatively favorite desirable food source for frugivory animals. Also, seeds of *A. eriantha* can germinate readily upon maturity and are potentially capable of establishing new populations.

The significant phylogeographic structure was supported by chloroplast data according to SAMOVA (Fig. 1), Hierarchical AMOVA and the comparison values of *N*_ST_ and *G*_ST_. Although three regional groups were identified by SAMOVA (Fig. 1) based on cpDNA, the two SAMOVA groups “Southeast edge” and “Southwest edge” probably represent areas of contact between haplogroups and may not reflect ‘true’ distinct biological units but mere artefacts due to low sample size. Thus, a closer inspection with nuclear DNA markers is needed. Five genetic clusters were revealed by the Bayesian assignment, PCoA and unrooted NJ tree analyses based on 31 neutral nuclear SSR loci (Fig. 4). Five clusters are roughly consistent with the east-west distribution of the species (Fig. 4b), demonstrating good geographic coherence. It is not uncommon that nuclear DNA markers can provide the high resolution required to explore the genetic structures of intra- or interspecies, for example in *Noccaea caerulescens* (J. Presl & C. Presl) F. K. Mey [34] and *Betula* genus [35].

### The refugium along the oceanic–continental gradient and the ‘oceanic’ adaptation

Both SDM and molecular phylogeography results revealed that the Zhejiang-Fujian Hilly Region next to the East China Sea is a refugium of *A. eriantha*. The Zhejiang-Fujian Hilly Region is always included in the predicted potential distribution areas of the species during the four glacial/interglacial periods in SDM (Fig. 6). In molecular analyses, the BBM results based on the cpDNA indicated that *A. eriantha* spread from its eastern ancestral area to other distribution areas (Additional file 3) and the ABC analysis based on nuclear SSRs showed that Cluster I, II and IV in the WD of *A. eriantha* originated from Cluster V in the ED (Fig. 5). The high haplotype richness based on cpDNA (Fig. 1a, Additional file 1) and high genetic diversity based on neutral nuclear SSRs (Fig. 3, Additional file 5) in the Hilly Region are consistent with the expectation that repeated contraction and expansion to and from refugia leave genetic signals of high diversity in refugial areas and low diversity in areas of expansion [36, 37]. Higher levels of historical gene flow from Cluster V to Cluster I (0.082) and Cluster IV (0.169) unidirectionally also imply that the Zhejiang-Fujian Hilly Region is a refugium of *A. eriantha*.

The cpDNA results (Fig. 1 and Additional file 3) revealed that HA and NJ with Haplotypes H19-H22 in the southern part of the Zhejiang-Fujian Hilly Region is the ancestor of three populations LP, CB and DK with Haplotype H23 on/around the Xuefeng Mountains, implying expansion from HA and NJ to the west distribution range. However, the haplotypes in the two populations are highly divergent from other haplotypes present in the Zhejiang-Fujian Hilly Region refugium. The localised highly divergent populations (Fig. 1b) indicate persistent isolation with other populations in the refugium from middle Pleistocene with no further expansion [38]. Thereby the populations have not been the source of major glacial recolonization as demonstrated by the ABC analysis based on neutral nSSRs (Fig. 5 and Additional file 9). The phylogeographic lineages remain geographically distinct within the refugium, which fit the ‘refugia within refugia’ pattern that has been described in the Iberian [39]. Such pattern revealed the internal complexity of the Zhejiang-Fujian Hilly Region as a refugium along the oceanic–continental gradient.

The preference for higher humidity during the coldest quarter and lower seasonal temperature difference of *A. eriantha* and its dispersal along the longitude imply a refugium along the oceanic–continental gradient and ‘oceanic’ adaptation of the species. SDM analysis indicated that Precipitation of Coldest Quarter (BIO19; importance of 44%), Temperature Seasonality (BIO4; 29%) and Mean Temperature of Wettest Quarter (BIO8; 25%) contributed the most to model development. By checking the isolines in the three climate layers during the four glacial/interglacial periods, we found that *A. eriantha* prefers higher precipitation during the coldest quarter, lower seasonal temperature difference and lower mean temperature during the wettest quarter (Additional file 11). The precipitation during the coldest quarter is similar to that of the present (Additional file 11), despite the climate of this region during the LGM (last glacial maxima) being dryer by c. 400–600 mm/yr [18, 19]. According to the location and type of the refugium of *A. eriantha*, we inferred that some or at least one of the populations of *Actinidia* fragmented in the Zhejiang-Fujian Hilly Region adapted ‘oceanic’ climate and formed *A. eriantha* during late-Miocene/early-Pliocene. Although a dipole-type circulation pattern of atmosphere during winter caused by the Tibetan Plateau and a lag time in spring warming between land and sea during February generate the moist environment in the coldest season in the east subtropical China [40–42], the moist level should always decrease from the coast to inland areas during glacial cycles as ranges of mountains in the Zhejiang-Fujian Hilly Region intercept clouds and cause major local increase in rain and snowfall. Then the species expand from the refugium repeatedly with the higher moist during coldest quarter and lower seasonal temperature difference during wettest quarter during the glacial periods.

### The biogeographic history of *Actinidia eriantha*

*Actinidia eriantha* in the refugium dispersed westward along a continuously east-west dispersal route from the Zhejiang-Fujian Hilly Region next to the East China Sea (Additional file 3, Figs. 5, 6 and 7). The corridor role of the Wuyi, Nanling, and Luoxiao Mountains during the late Quaternary has been previously mentioned [8, 13].

*Actinidia eriantha* went through repeated glacial expansions. The BBM analysis based on cpDNA estimated six dispersal and six subsequent vicariance events in the species. The BSP, MDA and neutrality tests based on cpDNA confirmed the indication of possible expansions in Lineage 2 by the star-shaped haplotype TCS network based on cpDNA (Fig. 1). The BSP showed that a slight population expansion occurred between c. 3.5 and 1.5 Ma (Fig. 2a). Based on neutral nuclear SSRs, DIYABC revealed that expansions along with the divergence of the five genetic clusters happened between 3.22 and 1.00 Ma, which is close to the expansion period estimated in BSP based on cpDNA. The two genetic data sets used here provide signals of demographic events which have occurred at the same time scales. The result of MDA showed that populations went through a sudden expansion 81,775 yr BP (95% CI: 10,112-95,404), which coincides with the early part of the last glacial period over the past 800,000 years (c. 115,000 – c. 11,700 years ago) [43]. Additionally, *A. eriantha* underwent a significant range expansion during the LGM (21 kya BP) based on SDM. Accumulating evidence suggests glacial expansion was not a rare event in subtropical China (e.g. [12, 44]).

The more highly fragmented distribution area of the species is evident from the LGM to the future based on SDM (Fig. 6). This is supported by lower contemporary gene flow among most clusters than historical gene flow (Additional file 7) suggesting that gene flow among these clusters is now more restricted.

### Important roles of geographic distance and environment distance for intraspecific divergence

Both geographic distance and environment distance have important roles for intraspecific divergence of *A. eriantha*. Environment effects can contribute to patterns of genetic structure of species either genome-wide or only in particular genomic regions [45]. Environment factors played an important role in adaptive differentiation of *A. eriantha* since the result of the identity test supports the existence of niche differentiation between the two groups (Fig. 6 and Additional file 13). Contribution analysis of SDM revealed both precipitation and temperature made great contributions to model development. In addition, the divergence times of the two lineages detected in *A. eriantha* based on three cpDNA fragments were dated to 4.03 Ma (95% HPD: 2.47-5.57 Ma) (Fig. 1b), which coincide with a turning point of drastic climate fluctuation during the middle Pliocene [46]. However, environment distance has no significant correlation with genetic differentiation of *A. eriantha* based on neutral loci (Table 1), suggesting that the effects of environment factors have not yet spread genome-wide. The predicted patterns for IBD are that genetic differentiation at neutral loci increases with increasing geographic distance, as a consequence of reduced gene flow as geographic distances increase [47]. The pattern has been proved by the IBD test (*β*_*D*_ = 0.279, *P* < 0.001) (Table 1).

## Conclusions

In summary, this is the first integrated evidence of a refugium of *Actinidia eriantha* along the oceanic–continental gradient and ‘oceanic’ adaptation of the species in the subtropical China. Molecular phylogeography and species distribution modelling revealed west-east geographically distinct divergence of *Actinidia eriantha*. Both geographic distance and climate difference all have important roles for intraspecific divergence of the species. The Zhejiang-Fujian Hilly Region was demonstrated to be a refugium along the oceanic–continental gradient and fit the ‘refugia in refugia’ pattern. After originating from the Zhejiang-Fujian Hilly Region with ‘oceanic’ adaptation, *A. eriantha* expanded to its western distribution range along the dispersal corridor repeatedly during the glacial periods. This study gives a deeper understanding of the refugia in subtropical China and will contribute to the conservation and utilization of kiwifruit wild resources since the identification of the refugium and the genetic diversity it harbour enables the refugium to be targeted for protection and collection in the context of climate change.

## Materials and methods

### Sample collection and DNA extraction

The leaves of 629 individuals from 28 locations were sampled across the entire distribution range of *A. eriantha* (Table 2, Fig. 1). Nine to 35 individuals in each population were sampled, except for populations CB and GD on the periphery of the range, in which only five and eight individuals were found, respectively. Three individuals of *A. fulvicoma* and one individual of *A. chinensis* were used as outgroups. Fresh leaves of outgroup species were collected from the Wuhan Botanical Garden, CAS. Voucher specimens representative of all samples are stored at the Herbarium of Wuhan Botanical Garden. Total genomic DNA was extracted from silica-dried leaves using a modified CTAB method [48]. Quality and concentration of the DNA were confirmed using 1% agarose gel electrophoresis and NanoDrop 8000 (Thermo Fisher Scientific, Waltham, MA, USA).

**Table 2:** Characteristics of sampled populations of *Actinidia eriantha*

Population code	Location	Longitude (E)	Latitude (N)	Altitude (m)	Sample size
WH	Wuhua County, Guangdong Prov.	115°23′	23°52′	686	28
LY	Luoyuan County, Fujian Prov.	119°24′	26°27′	529	35
DH	Dehua County, Fujian Prov.	118°11′	25°40′	1001	30
JG	Mount Jinggang, Jiangxi Prov.	114°11′	26°36′	1000	33
LiS	Lishui city, Zhejiang Prov.	119°46′	28°15′	365	33
SQ	Mount Sanqing, Jiangxi Prov.	118°03′	28°12′	589	34
AY	Anyuan County, Jiangxi Prov.	115°23′	25°00′	433	29
RY	Ruyuan County, Guangdong Prov.	113°03′	24°57′	820	33
RJ	Ruijin City, Jiangxi Prov.	116°13′	25°56′	400	30
NJ	Nanjing County, Fujian Prov.	117°12′	24°53′	695	10
WC	Wencheng County, Zhejiang Prov. ProvProvince	119°52′	27°30′	400	35
XF	Xinfeng County, Guangdong Prov.	113°03′	24°57′	594	25
CY	Chongyi County, Jiangxi Prov.	114°14′	25°38′	478	21
YM	Mount Yangming, Hunan Prov.	111°56′	26°7′	1179	15
SH	Mount Shunhuang, Hunan Prov.	111°00′	26°24′	668	9
JH	Jianghua County, Hunan Prov.	111°42′	25°13′	420	15
LS	Mount Lu, Jiangxi Prov.	115°58′	29°33′	1080	20
WY	Mount Wuyi, Jiangxi Prov.	117°43′	27°20′	500	33
LC	Lichuan County, Jiangxi Prov.	116°50′	27°05′	277	30
ShQ	Shiqian County, Guizhou Prov.	108°08′	27°20′	977	12
LP	Liping County, Guizhou Prov.	109°25′	26°06′	450	20
CB	Chengbu County, Hunan Prov.	110°09′	26°22′	1485	5
DK	Dongkou County, Hunan Prov.	110°40′	27°14′	535	9
GD	Guiding County, Guizhou Prov.	107°03′	26°15′	1113	8
YP	Yuping County, Guizhou Prov.	108°52′	27°09′	506	21
QY	Qiyang County, Hunan Prov.	112°06′	26°15′	146	15
WGS	Mount Wugong, Jiangxi Prov.	114°13′	27°29′	619	22
HA	Hua’an County, Fujian Prov.	117°26′	24°52′	861	20

### Laboratory protocols

For the phylogeographic cpDNA analysis, we sequenced three non-coding intergenic spacer (IGS) regions: *ndhF*-*rpl132* (F: ACAGGAACTGGAAGTGGAACAA; R: TTGGTCAAGGTCGAGGAAAGAA), *rps*16-*trn*Q (F: GTCGCACGTTGCTTTCTACC; R: TAGCTGCGTTGTCCGAATCT), and *trn*E-*trn*T (F: ATACTTGCCCGACCGACATC; R: GAACCGACGACTTACGCCTT). The cpDNA of *A. eriantha* is inherited paternally [49]. In total 221 individuals from 28 populations of *A. eriantha*, three individuals of *A. fulvicoma* and one of *A. chinensis* were sequenced. Polymerase Chain Reactions were conducted in 20 μl reactions containing 10 μl 2× *Taq* PCR MasterMix (Bioteke), 0.5 μl each primer (0.2 μM), 1 μl template DNA (ca. 50-100 ng) and 8 μl ddH_2_O. Thermo-cycling conditions were as follows: 94 °C for 4 min; 35 cycles of 94 °C for 30 s, 58 °C for 60 s, and 72 °C for 60 s; a final 10 min extension at 72 °C. Sequences were generated with an ABI 377XL DNA sequencer (Applied Biosystems).

For nuclear DNA analysis, 629 individuals were genotyped at 38 nuclear microsatellite loci (Additional file 4) with 32 EST-SSRs described by Guo et al. [31] and six nuclear SSRs screened from *A. chinensis* (five genomic SSRs: UDK96-030, UDK96-026, UDK96-040, 751, 761 [50, 51] and one EST-SSRs [52]). Amplification of SSR loci followed the protocol in Guo et al. [31]. Fluorescently labelled PCR products were spiked with the internal size standard GeneScan 500 LIZ and separated on a 3730xl DNA Analyzer (Applied Bio systems). Alleles were scored manually in GeneMarker v. 2.2 (SoftGenetics, Pennsylvania, USA). Microsatellite quality was checked using MSAnalyser [53].

### Data analysis

#### Analysis of cpDNA variation

All sequences were checked using Finch TV v. 1.4 (https://digitalworldbiology.com/FinchTV). Three cpDNA regions were aligned and trimmed separately in MEGA v. 6 [54] and combined using FaBox v. 1.5 (https://users-birc.au.dk/~palle/php/fabox/index.php). Haplotype (*h*) and nucleotide (π) diversity were calculated for each population and the overall species using DnaSP v. 5 [55]. Two parameters for population differentiation (*G*_ST_, *N*_ST_) were analyzed using the program PermutCpSSR v. 1.2 (http://www.mybiosoftware.com/tag/permutcpssr).

To infer possible regional groups of populations, spatial analysis of molecular variance (SAMOVA) of cpDNA was implemented in SAMOVA v. 1.0 [56]. The most likely number of groups (*K*) was determined by repeatedly running the program SAMOVA with 2–28 groups and choosing those partitions with a maximum value of differences among groups of populations (F_CT_). Hierarchical analysis of molecular variance (AMOVA) [57] was also conducted in Arlequin v. 3.5 [58] to quantify the proportion of total genetic variance explained by differences between regional population groups (as identified by SAMOVA) and between populations within groups. Significance of variance components was tested with 10, 000 permutations.

Genealogical relationships of identified haplotypes were inferred from a 95% statistical parsimony network constructed in TCS v.1.2 [59]. To identify cpDNA lineages and estimate divergence time among lineages, we performed Bayesian phylogenetic inference on the haplotypes with a relaxed clock model in BEAST2 v. 2.4 [60]. A Yule prior was applied for the inter-species relationships and a coalescent prior assuming constant population size for the intra-species relationships. The GTR model was selected as the best-fit substitution model of molecular evolution using jModelTest v. 2.1 [61]. The diversification in *Actinidia* has been previously elucidated [62]. Therefore, we can take advantage of the timing of kiwifruit diversification as calibration points when doing our dating analyses. Two calibration points were used with a normal distribution prior: the split between *A. chinensis* and the two other species of 11 Myr (95%CI: 4.7-17.3 Myr) and the split between *A. eriantha* and *A. fulvicoma* of 5.5 Myr (95%CI: 4.2-6.8 Myr) [62]. We did not use the calibration point of the split between *A. eriantha* and its closest relative *A. latifolia* since *A. latifolia* shows up in the ingroup of *A. eriantha* in the tree constructed with the three cpDNA markers. The length of the Markov Chain Monte Carlo (MCMC) algorithm was set to 1 billion generations, sampling every 100,000 generations with the first 20% discarded as burn-in. We checked the convergence of parameters using Tracer v. 1.6 [63]. The consensus tree was analyzed using TreeAnnotator and visualized in FigTree (http://tree.bio.ed.ac.uk/software/figtree/).

We used bayesian skyline plots (BSP) in BEAST2 v. 2.4 and the mismatch distribution analysis (MDA) in Arlequin v. 3.5 to detect population spatial expansion events. BSP was conducted with the same settings as in previous analyses except that the priors setting was changed to Coalescent Bayesian Skyline. BSP has the advantage of not assuming any demographic scenario a priori [64]. The goodness-of-fit under a sudden-expansion model was tested with the sum of squared deviations (SSD) and Harpending’s raggedness index (HRag) [65] for MDA. The MDA-derived spatial expansion parameter (τ) was converted into generation time (T) using the following equation: T = τ/2 *μ* [66], where *μ* is the neutral mutation rate of the entire cpDNA sequence per generation. The value for *μ* was calculated as *μ* = *u*k, where *u* is the substitution rate (here, 1.0 × 10^− 8^ substitutions/site/generation(s/s/g) [62], and k represents the sequence length of the cpDNA region (here, 1605 bp). Finally, the expansion time was calculated assuming a generation time of 7 years for *A. eriantha* under natural conditions [62]. We also calculated Tajima’s *D* [67] and Fu’s *F*_S_ [68] to assess possible expansions using Arlequin v. 3.5.

The ancestral areas were reconstructed using the BBM (Bayesian Binary MCMC) implemented in RASP v. 3.0 [69]. In total, 10,000 BEAST-generated trees and one consensus tree were used as input with outgroups removed and a 10% burn-in. Three regions (west, middle and east areas) were defined according to the precision of consensus tree and the distribution range of the species (Additional file 3). The F81 model (variable base frequencies, all substitutions equally likely) [70] was used according to Akaike Information Criterion (AIC: F81 = 4949, JC = 5335) estimated using jModelTest.

### Nuclear data analysis

To identify putative locally adaptive loci that may affect analysis of neutral population structure and demographic history, we used two methods: genetic-environment association analysis in SamBada v. 0.5.3 [71] and differentiation outlier in Arlequin with a significance threshold of 0.01 corrected using the Bonferroni method. These two methods have recently become widely used to test for loci under selection [72]. A total of 19 bioclimatic variables (http://www.bioclim.org) served as environmental variables. Two sets of loci were obtained: loci under selection and neutral loci. A locus was considered to be under selection if both outlier tests showed significance. The correlation between the outlier loci and environment factors was checked using SamBada. Gene Ontology (GO) annotations [73] of the sequences containing the outliers were conducted. A locus was considered to be neutral if both tests showed no significance. Successive nuclear data analyses were based only on neutral loci except for the analysis of characterization of each locus.

Polymorphism of each microsatellite locus, i.e. the number of observed alleles (*N*_A_), the observed (*H*_O_) and the excepted heterozygosity (*H*_E_), and polymorphism information content (PIC) was calculated using CERVUS v. 3.0 [74]. The mean fixation coefficient *F*_IS_ was calculated using FSTAT v. 2.9 [75]. Significant deviation from Hardy-Weinberg equilibrium (HWE) induced by heterozygote deficiency was tested across all populations and each population-locus combination using 10,000 permutations in GENEPOP v. 4.2 (http://genepop.curtin.edu.au/). Significance was corrected for multiple testing using the Bonferroni method. To account for any departure from HWE due to the presence of null alleles in each locus, we estimated their frequency within each population in FreeNA with a cutoff value of 0.2 [76]. Linkage disequilibrium among the 38 loci was evaluated in GENEPOP for each population-locus-locus combination using 10,000 permutations.

Intrapopulation genetic variation was estimated by the total number of alleles (*A*_T_), the number of private alleles (*A*p), the effective number of alleles (*A*e), the observed (*H*_O_) and the excepted heterozygosity (*H*_E_), unbiased expected heterozygosity (U*H*_E_) [77], and the inbreeding coefficient *F*_IS_ across 31 neutral loci using GeneAlex v. 6.5 [78]. The allele richness (*R*_S_, standardized for four individuals using rarefaction) was calculated using FSTAT. The two genetic parameters U*H*_E_ and *R*_S_ were used for removing the bias caused by the strong variation in the sample size. Genetic diversity based on standardized *A*e and U*H*_E_ were visualized as genetic landscapes in ArcGIS v. 10.2 (Environmental Systems Research Institute, Inc., Redlands, CA), using the Genetic Landscapes Toolbox [79]. Significant deviations of Hardy-Weinberg equilibrium induced by heterozygote deficiency were tested in GENEPOP for each population across 31 neutral loci using 10,000 permutations. Pairwise population *F*_ST_ was estimated using GeneAlex. Genetic distance based on standardized *F*_ST_ was visualized as divergence landscape.

Several methods were used to investigate the genetic structure of *A. eriantha*. A principal coordinate analysis (PCoA) of populations was performed in GenAlex and the first two PC axes (PC1 and PC2) were used to explore the genetic relationships among populations. The genetic relationships were also evaluated by generating a neighbor-joining (NJ) network based on *D*_A_ distances [80] using POPTREE v. 2 [81]. The most likely number of clusters was inferred by Bayesian clustering implemented in STRUCTURE v. 2.3 [82]. Assuming admixture and independent allele frequencies, we set up a run with a burn-in period of 100,000 iterations followed by 500,000 recorded iterations for *K* = 1 to *K* = 28 clusters and 10 runs per *K* value. The most probable number of clusters was determined using the *ΔK* approach [83] in STRUCTURE HARVESTER [84].

Migrate-n v. 3.6 [85] was used to estimate levels of historical gene flow between each pair of clusters by calculating the mutation-scaled effective immigration rate (*M*) with the Brownian motion approximation. Three independent analyses were run, recording every 100 steps with 500,000 genealogies and a 10,000 genealogy burn-in. Immigration rate (*m*) was calculated as *m* = *Mμ* with *μ* being the mutation rate (estimated for nSSRs as 3 × 10^− 4^, [86]). BayesAss v. 3.0 [87] was used to estimate the contemporary counterpart by reckoning each pairwise *m*. A total of 5,000,000 MCMC iterations were run with 20% burn-in.

We used ABC simulations in DIYABC v2.0 [88] to determine the historic process involved in the settlement of clusters identified by STRUCTURE based on the 31 neutral SSR loci. Due to computational limitations and the infinite number of possible scenarios when numerous populations are considered, inferences were based on finite set of genetically and geographically delimited groups. According to the phylogeography results using of cpDNA, patterns of genetic divergence revealed by SSR, and SDM during past, present and future, we denoted clusters located in the eastern distribution (ED) range (Cluster V and Cluster III) as source genetic units and evaluated the relationship among the source genetic units. Furthermore, we detected the origin of each colonization genetic unit located in the western distribution (WD) range, and the order of divergence of the units. Scenarios were evaluated in a three-step analysis. First, a set of scenarios aimed to analyze the relationship among the source genetic units (set A). Second, based on the results from set A, three sets of scenarios were designed to analyze which source genetic unit Cluster I, II or IV diverged from (set B, C and D). Finally, Set E was calculated to test the relationship among colonization genetic units and their source genetic units, based on the results yielded by set B, C and D. A list of all parameters and prior distributions used to model scenarios is summarized in Additional file 8. The type I and mean type II error for the most likely scenarios were calculated to estimate the statistical power [89]. The time parameters were estimated in generations. As colonization may generate strong demographic bottleneck leading reduction in genetic diversity, times of demographic bottleneck were considered in our set of scenarios.

### Isolation by distance and isolation by environment analyses based on both neutral SSR loci and cpDNA dataset

To evaluate the effect of geographic and environmental conditions on genetic divergence, we tested for isolation by distance (IBD) and isolation by environment (IBE) based on neutral SSR loci and cpDNA dataset. IBE based on potentially adaptive loci was not tested since only one outlier locus was obtained. Two methods were used: multiple matrix regression with randomization (MMRR) [90] using the *R* function ‘MMRR’ and partial Mantel test in the package vegan v. 2.4 [91]. We used pairwise *F*_*ST*_/(1-*F*_*ST*_) [92] to represent population pairwise genetic distances. Geographic distances were shown by the log10 of geographic distances between pairs of populations. For environmental variables, we obtained 19 bioclimatic variables (www.worldclim.org). To reduce bioclimatic covariance, principal component analysis (PCA) was conducted using the R package vegan. The first three PC axes (PC1, PC2 & PC3) explaining > 89% of the total variation were used to compute Climatic (Euclidian) distance.

### Species distribution models and dispersal corridors analyses

To infer the potential distribution areas of *A. eriantha* across a complete glacial-interglacial cycle, 168 occurrence points of *A. eriantha* including 28 sampled locations, six location studied by Liu et al. [29] and 134 herbaria specimens (Additional file 12) from the Chinese Virtual Herbarium (http://www.cvh.ac.cn/) were used to compute species distribution models (SDM) in Maxent v. 3.2 [93] based on Future (2080), Current (1950-2000), LGM (21 kya BP), and LIG (120-140 kya BP) climatic maps (http://www.worldclim.org). Model performance was evaluated using the area under the Receiver Operating Characteristic Curve (AUC). To avoid over-fitting, we selected five bioclimatic variables (|r| > 0.8): BIO2 (Mean Diurnal Range), BIO4 (Temperature Seasonality), BIO8 (Mean Temperature of Wettest Quarter), BIO16 (Precipitation of Wettest Quarter) and BIO19 (Precipitation of Coldest Quarter), based on correlation analysis using ENMTools v. 1.3 [94] and their contributions to SDM. Relative contributions of the environmental variables to model development were estimated in Maxent. Model predictions were visualized in DIVA-GIS (http://www.diva-gis.org/gdata). To determine whether the WD and ED part of the species occupy identical climatic environments (‘niches’), we performed SDM in Maxent and niche identity tests in ENMTools. A one-tailed *t*-test was used to estimate the significant difference between observed values of Warren et al.’s *I* [95] and Schoener’s *D* [96] and 100 randomized distributions of *D* and *I* generated by randomly assigning samples to either part.

Dispersal corridors at the current time, LGM, and LIG were mapped by applying the CLCP method and SDM toolbox [97] in ArcGIS. To create the ecological dispersal network, we converted SDM generated above to dispersal cost layers. Chloroplast haplotype pairwise population networks were generated by taking the sum of the least cost paths (LCPs) among all shared and sister haplotypes from different localities in ArcGIS. Standardized dispersal corridor layers of *A. eriantha* were established using SDM toolbox.

## Supplementary Information

**Additional file 1. **Genetic characteristics of cpDNA in 28 *Actinidia eriantha* populations.

**Additional file 2. **Analysis of molecular variance (AMOVA) for 28 sampled populations of *Actinidia eriantha* based on cpDNA.

**Additional file 3.** The ancestral areas were reconstructed using the BBM (Bayesian Binary MCMC) method. (a) The ancestral areas were reconstructed using the BBM implemented in RASP v. 3.0. Three regions (west, middle and east areas) were defined according to the precision of consensus tree and the distribution range of the species. (b) Geographical locations of the three regions and the most likely dispersal direction.

**Additional file 4.** Characteristics of 38 nuclear microsatellite loci.

**Additional file 5.** Genetic diversity of each population based on 31 neutral nuclear microsatellite loci.

**Additional file 6. **The most probable number of clusters determined using the Delta *K* approach. (a) When *K* = 2 for all individuals, Delta *K* has the highest value. (b) When *K* = 4 for western subset (plus HA and NJ) of individuals, Delta *K* has the highest value.

**Additional file 7. **Migration rates (*m*) across the five clusters of *Actinidia eriantha* based on 31 neutral nuclear SSRs.

**Additional file 8.** Prior distributions of the parameters used in DIYABC.

**Additional file 9. **Set A, B, C and D of scenarios in DIYABC of *Actinidia eriantha*. Posterior probability of each scenario obtained by logistic regression of 1% of the closest simulated datasets is shown on the top of the scenario. Scenario outlined in red is the best option.

**Additional file 10.** Estimations of posterior distributions of parameters revealed by DIY-ABC for the best scenarios of set A, B, C, D and E, respectively.

**Additional file 11.** The isolines in the three climate layers Bio 4, Bio 8 and Bio 19.

**Additional file 12. **Information of 134 herbaria specimens of *Actinidia eriantha*.

**Additional file 13. **The results of niche identity test between West group and East group in subtropical China. The histogram indicate the randomized distributions of Warren et al.’s *I* and Schoener’s *D* and the arrow indicates the observed values of *I* and *D*. The x-axis indicates values of *I* and *D*, and the y-axis indicates the number of randomizations.

**Additional file 14.** cpDNA haplotype with its Gene Bank accession number.

## Data Availability

All haplotype sequences are deposited in GenBank (accession numbers: MN974299-974370; MW387118-387126) (Additional file 14).
