# Supplementary material for: Molecular phylogeography and species distribution modelling evidence of ‘oceanic’ adaptation for Actinidia eriantha with a refugium along the oceanic–continental gradient in a biodiversity hotspot
Source: BMC Plant Biol. 2022 Feb 28;22:89. doi: 10.1186/s12870-022-03464-5 (PMC8883688; doi:10.1186/s12870-022-03464-5)
Supplement: Supplementary file 1 — Additional file 1. Genetic characteristics of cpDNA in 28 Actinidia eriantha populations. [file 12870_2022_3464_MOESM1_ESM.docx]

| Additional file 1 Genetic characteristics of cpDNA in 28 *Actinidia eriantha* populations. | | | | |
| --- | --- | --- | --- | --- |
| Population code | n | Hd (±SD) | π×10^-3^ (±SD) | H (n) |
| AY | 8 | 0 | 0 | H1 (8) |
| CB | 5 | 0.60 (±0.18) | 2.78 (±0.81) | H1 (3), H23 (2) |
| CY | 8 | 0.54 (±0.12) | 0.35 (±0.08) | H1 (3), H17 (5) |
| DH | 8 | 0 | 0 | H1 (8) |
| DK | 8 | 0.25 (±0.18) | 1.16 (±0.83) | H1 (7), H23 (1) |
| GD | 8 | 0 | 0 | H1 (8) |
| HA | 8 | 0.75 (±0.10) | 0.64 (±0.12) | H20 (3), H21 (3), H22 (2) |
| JG | 8 | 0 | 0 | H1 (8) |
| JH | 8 | 0 | 0 | H1 (8) |
| LC | 8 | 0.75 (±0.14) | 0.71 (±0.17) | H1 (2), H12 (4), H13 (1), H14 (1) |
| LiS | 8 | 0 | 0 | H1 (8) |
| LP | 8 | 0.25 (±0.18) | 1.16 (±0.83) | H1 (1), H23 (7) |
| LS | 8 | 0 | 0 | H1 (8) |
| LY | 8 | 0 | 0 | H1 (8) |
| NJ | 8 | 0.68 (±0.12) | 1.11 (±0.18) | H1 (4), H19 (1), H20 (3) |
| QY | 8 | 0 | 0 | H1 (8) |
| RJ | 8 | 0.43 (±0.17) | 0.28 (±0.11) | H1 (6), H16 (2) |
| RY | 8 | 0.54 (±0.12) | 0.35 (±0.08) | H1 (3), H2 (5) |
| SH | 8 | 0.43 (±0.17) | 0.28 (±0.11) | H1 (6), H18 (2) |
| ShQ | 8 | 0 | 0 | H1 (8) |
| SQ | 8 | 0.75 (0.14) | 0.78 (±0.25) | H8 (4), H9 (2), H10 (1), H11 (1) |
| WC | 8 | 0.46 (±0.20) | 0.33 (±0.16) | H1 (6), H6 (1), H7 (1) |
| WGS | 8 | 0 | 0 | H1 (8) |
| WH | 8 | 0.61 (±0.16) | 0.52 (±0.17) | H3 (5), H4 (2), H5 (1) |
| WY | 8 | 0.25 (±0.18) | 0.17 (±0.12) | H1 (7), H8 (1) |
| XF | 8 | 0 | 0 | H1 (8) |
| YM | 8 | 0 | 0 | H1 (8) |
| YP | 8 | 0.57 (±0.094) | 0.38 (±0.06) | H1 (4), H15 (4) |
| n, number of sampled individuals; Hd, haplotype diversity; π, nucleotide diversity; h, haplotype. | | | | |
