# Supplementary material for: Molecular phylogeography and species distribution modelling evidence of ‘oceanic’ adaptation for Actinidia eriantha with a refugium along the oceanic–continental gradient in a biodiversity hotspot
Source: BMC Plant Biol. 2022 Feb 28;22:89. doi: 10.1186/s12870-022-03464-5 (PMC8883688; doi:10.1186/s12870-022-03464-5)
Supplement: Supplementary file 3 — Additional file 3. The ancestral areas were reconstructed using the BBM (Bayesian Binary MCMC) method. (a) The ancestral areas were reconstructed using the BBM implemented in RASP v. 3.0. Three regions (west, middle and east areas) were defined according to the precision of consensus tree and the distribution range of the species. (b) Geographical locations of the three regions and the most likely dispersal direction. [file 12870_2022_3464_MOESM3_ESM.pdf]

(a)

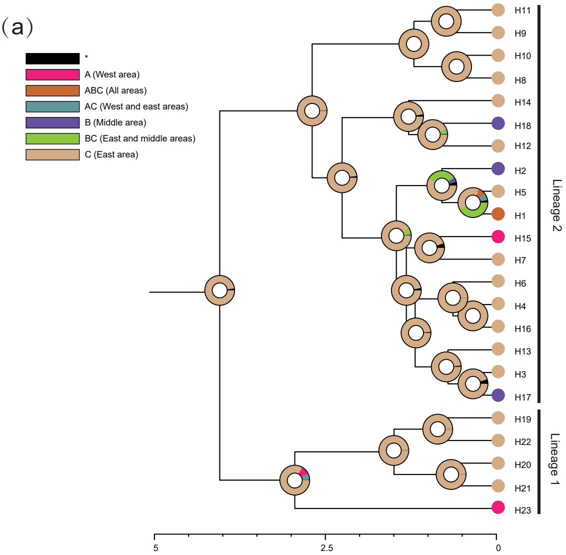


(b)

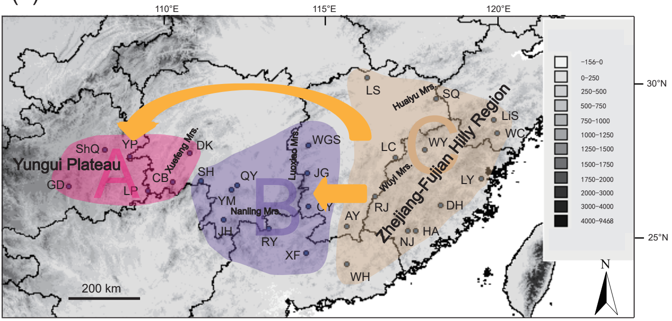


Additional file 3. The ancestral areas were reconstructed using the BBM (Bayesian Binary MCMC) method. (a) The ancestral areas were reconstructed using the BBM implemented in RASP v. 3.0. Three regions (west, middle and east areas) were defined according to the precision of consensus tree and the distribution range of the species. (b) Geographical locations of the three regions and the most likely dispersal direction.
