# Supplementary material for: Molecular phylogeography and species distribution modelling evidence of ‘oceanic’ adaptation for Actinidia eriantha with a refugium along the oceanic–continental gradient in a biodiversity hotspot
Source: BMC Plant Biol. 2022 Feb 28;22:89. doi: 10.1186/s12870-022-03464-5 (PMC8883688; doi:10.1186/s12870-022-03464-5)
Supplement: Supplementary file 4 — Additional file 4. Characteristics of 38 nuclear microsatellite loci. [file 12870_2022_3464_MOESM4_ESM.docx]

| Additional file 4 Characteristics of 38 nuclear microsatellite loci. | | | | | | | | | |
| --- | --- | --- | --- | --- | --- | --- | --- | --- | --- |
| Locus | Production | SSR | Forward Primer | Reverse Primer | *N*A | *H*O | *H*E | PIC | HEW |
| UDK96-030 | 115 | (AG)9(AC)15 | TCATGTTTGTGGTTGAGTTGTG | AGCAATAAACTCAAGCGCGT | 28 | 0.687 | 0.909 | 0.901 | ND |
| UDK96-026 | 173 | (AC)14 | CGCTGACCAGATTCTGATGA | TTGAAAATCACTGAGCACAACC | 21 | 0.471 | 0.785 | 0.773 | ND |
| 751 | 187 | (CT)8 | GCAAGCCCTAAATTCAATAG | ATCTCCACCGAACTTGGCCG | 15 | 0.461 | 0.83 | 0.808 | ND |
| UDK96-040 | 177 | (AC)15(AG)21 | TCGAGTTACCTAGCTACTCCGC | CAAGGGAAGAAAATGTTGAACC | 31 | 0.56 | 0.938 | 0.933 | ND |
| Ke221 | 75-200 | (AT)16 | TGAGTTGTGGGTATTGCAAGTT | GCAGCAGTGCTAAACCTGTG | 7 | 0.312 | 0.625 | 0.591 | ND |
| AET180 | 148 | (TTTC)5 | TCAAATAACTGCGCCTGTTG | GCTCAATCGAGGGTAATGGA | 5 | 0.348 | 0.611 | 0.535 | ND |
| 761 | 152 | (CT)2CC(CT)8 | GATTTGCTGTTTGTAAGGCC | ATCCGACTTCCAAGCCTCTC | 24 | 0.607 | 0.899 | 0.89 | ND |
| AET81 | 225 | (TTG)7 | ATCGAAGTGGGTTCTTGGTG | TTGCGTAGCTTAGCTGTAGGC | 11 | 0.52 | 0.758 | 0.719 | ND |
| AET28 | 158 | (ATAC)5 | GCCAAAGCATCTCCCATAAA | TCGCTCATCTCTCTCTCGGT | 7 | 0.456 | 0.753 | 0.714 | ND |
| AET9 | 191 | (CTAT)5 | AACTGGACTTGAATGCCGAC | TCGATCTCGAGCAGATGTTTT | 4 | 0.208 | 0.357 | 0.338 | *** |
| AET121 | 230 | (TCG)8 | ATCCTCGTCGTCATCTACCG | TGAAAACCATGGAGGAGGAG | 13 | 0.634 | 0.758 | 0.736 | *** |
| AET141 | 162 | (AAGA)5 | TCAGGAAGAAAGAGAGACTTTTGA | ACTTCCGGACATCCCTCTCT | 12 | 0.628 | 0.834 | 0.813 | ND |
| AET144 | 205 | (AGG)7 | CCCAACTCTGCCTGAAGAAG | CCTTGTTTGTCACTCGGTCA | 11 | 0.642 | 0.793 | 0.761 | *** |
| AET22 | 235 | (GTTT)5 | TCAGCAGCCATAAGTTTGGA | GGGGAGTGAACTTAGTTTGCC | 12 | 0.423 | 0.676 | 0.649 | ND |
| AET169 | 182 | (ATCC)5 | CAAAACGAGAAAGAAAGATATTCAC | GAAACCCTAACCCCCAAAAA | 6 | 0.145 | 0.293 | 0.281 | ND |
| AET167 | 224 | (ATAA)5 | TCGAGCGTATTTGGTGAAAA | CTATGGCTTGGAACACAGCA | 7 | 0.309 | 0.719 | 0.669 | ND |
| AET122 | 264 | (CCG)7 | GCGCTTAAGTCCTCTTTCCC | CAAACGCTCTTCCTCCTCTG | 9 | 0.493 | 0.76 | 0.721 | ND |
| AET82 | 183 | (TAGA)5 | TCCTCTTTGAAATGGCTTGC | CTTCTCGCCAAGACTTCGAC | 6 | 0.256 | 0.405 | 0.374 | *** |
| AET38 | 225 | (TGG)7 | GGGGACTAGGGATGGAGAAG | TGGGATTCCTTTCATTCTGC | 6 | 0.361 | 0.723 | 0.672 | ND |
| AET104 | 277 | (TGCT)5 | CTCCTGTTGGTTACGGGAAA | CGCATAGAACATCATCCGAA | 9 | 0.499 | 0.715 | 0.669 | *** |
| AET53 | 101 | (ATCT)5 | TTCCATTTCGCTTTCCATTC | ACCACCGCTCAATAATCTGG | 7 | 0.441 | 0.619 | 0.58 | ND |
| AET170 | 154 | (TTTG)5 | CAAGATCAAAGACTGTAAAATGGA | CATGCTCACCAAAGACCTAACA | 7 | 0.347 | 0.387 | 0.357 | ND |
| AET2 | 228 | (TG)10 | AAATATTCCCTAATTTTCTCTGTATCA | ACAGGACCTTCCTGCTCTCA | 21 | 0.708 | 0.912 | 0.905 | *** |
| AET97 | 110 | (CCA)7 | CGACACTGTCAAACCACACC | GAGAATGGAAGTGGATGGGA | 7 | 0.377 | 0.545 | 0.464 | *** |
| AET89 | 157 | (CAG)7 | CATTGGCTGCTTCGACTACA | ACCAAACCACCCTCCTTCTT | 19 | 0.592 | 0.812 | 0.798 | *** |
| AET171 | 232 | (AT)10 | TTTCCAGGGGGAGCTTTTAT | TTTGCAAAATTGGCTCTGAA | 20 | 0.335 | 0.847 | 0.834 | ND |
| AET76 | 116 | (CA)10 | TGGTAAACTCTTGCTGGCCT | TGAGCCCAAAATAGGTGGTC | 17 | 0.47 | 0.734 | 0.711 | ND |
| AET60 | 173 | (GA)10 | GGATGATCTGTTCCAGCGTT | TTGATTGGGTTTCAAGAGGG | 14 | 0.636 | 0.868 | 0.854 | ND |
| AET106 | 250 | (AT)10 | GGACTTCGACACCTTGGAAA | CCTTTCATCTCTCAAATCCCA | 32 | 0.346 | 0.905 | 0.897 | ND |
| AET160 | 118 | (TCT)7 | TCCTTTAGCGGGATCATACG | TGGGAACCTTCCTACAGGTG | 15 | 0.455 | 0.749 | 0.723 | ND |
| AET165 | 181 | (AGAA)5 | GCAAATAAAACAAGACTTCTAGTGGA | GTCCTCGACAGGATTGCCTA | 7 | 0.192 | 0.535 | 0.496 | ND |
| AET73 | 254 | (CAAA)5 | GGAACGAACCCACCAATATG | CGTGCGAGTCTAATTCCGTT | 6 | 0.351 | 0.613 | 0.541 | ND |
| AET178 | 136 | (ATAA)5 | AGGGGCTGTTTGTGTGTTTC | TTGGATATGAGGGCCTTGTT | 6 | 0.181 | 0.432 | 0.398 | ND |
| AET33 | 186 | (GCTG)5 | AGAGAGAGAGGAGGGTTGTGC | CGCAGTAGACGGCTACACTTC | 9 | 0.651 | 0.751 | 0.709 | *** |
| AET7 | 266 | (GGC)7 | GTATTTGGGGGTTTGGGATT | GCAGATTTCTGCTTCGCTCT | 13 | 0.426 | 0.771 | 0.744 | ND |
| AET48 | 139 | (GTG)7 | TTTCTGTTGAGGATCTGGGC | TTTGAAATGCATCTGAAGCG | 9 | 0.652 | 0.72 | 0.691 | ND |
| AET46 | 219 | (TATG)5 | ACAAAATTCGTTTGGCTTGG | CTCGCTATGCTGCCATTTGT | 11 | 0.447 | 0.615 | 0.537 | *** |
| AET130 | 279 | (TG)10 | GAGCTTGTAACGGCCTTCTG | ATTTCGGAAAATCACGCAAA | 19 | 0.605 | 0.825 | 0.802 | ND |

Notes: *N*_A_: the number of observed alleles, *H*_O_: the observed heterozygosity, *H*_E_: the excepted heterozygosity, PIC: polymorphism information content, HWE: Hardy-Weinberg equilibrium, ND: no significant difference.
