# Supplementary material for: Molecular phylogeography and species distribution modelling evidence of ‘oceanic’ adaptation for Actinidia eriantha with a refugium along the oceanic–continental gradient in a biodiversity hotspot
Source: BMC Plant Biol. 2022 Feb 28;22:89. doi: 10.1186/s12870-022-03464-5 (PMC8883688; doi:10.1186/s12870-022-03464-5)
Supplement: Supplementary file 5 — Additional file 5. Genetic diversity of each population based on 31 neutral nuclear microsatellite loci. [file 12870_2022_3464_MOESM5_ESM.docx]

| Additional file 5 Genetic diversity of each population based on 31 neutral nuclear microsatellite loci. | | | | | | | | |
| --- | --- | --- | --- | --- | --- | --- | --- | --- |
| Population | Individuals | *A*_T_ | *A*p | *A*e | *R*s | *H*o | U*H*e | *F*_IS_ |
| WC | 35 | 155 | 4 | 2.88 | 3.133 | 0.483 | 0.598 | 0.168** |
| SQ | 33 | 100 | 3 | 1.89 | 2.168 | 0.342 | 0.376 | 0.106** |
| LY | 35 | 131 | 3 | 2.57 | 2.811 | 0.480 | 0.540 | 0.117** |
| LC | 30 | 148 | 1 | 2.65 | 2.923 | 0.466 | 0.534 | 0.095** |
| WY | 33 | 166 | 2 | 3.00 | 3.169 | 0.514 | 0.614 | 0.137** |
| LS | 20 | 107 | 4 | 2.36 | 2.649 | 0.493 | 0.509 | 0.012 |
| RJ | 30 | 169 | 3 | 3.01 | 3.212 | 0.533 | 0.611 | 0.100** |
| LiS | 33 | 168 | 3 | 3.11 | 3.191 | 0.519 | 0.597 | 0.148** |
| DH | 30 | 154 | 4 | 2.66 | 3.005 | 0.499 | 0.580 | 0.119** |
| NJ | 10 | 100 | 2 | 2.42 | 2.737 | 0.497 | 0.541 | 0.019 |
| HA | 20 | 111 | 2 | 2.16 | 2.509 | 0.369 | 0.432 | 0.135** |
| WH | 28 | 115 | 2 | 2.31 | 2.583 | 0.456 | 0.489 | 0.097** |
| XF | 25 | 118 | 3 | 2.26 | 2.517 | 0.416 | 0.462 | 0.053** |
| AY | 29 | 133 | 5 | 2.31 | 2.627 | 0.389 | 0.468 | 0.131** |
| JG | 33 | 188 | 2 | 3.02 | 3.155 | 0.446 | 0.556 | 0.160** |
| WGS | 22 | 152 | 2 | 2.98 | 3.050 | 0.478 | 0.547 | 0.131** |
| RY | 33 | 143 | 5 | 2.42 | 2.671 | 0.477 | 0.477 | 0.018* |
| CY | 21 | 161 | 1 | 3.06 | 3.240 | 0.464 | 0.595 | 0.236** |
| JH | 15 | 136 | 1 | 2.79 | 2.996 | 0.479 | 0.536 | 0.065** |
| YM | 15 | 100 | 0 | 2.13 | 2.399 | 0.432 | 0.431 | 0.009 |
| SH | 9 | 76 | 4 | 1.66 | 2.014 | 0.326 | 0.324 | -0.018 |
| CB | 5 | 88 | 1 | 2.16 | 2.677 | 0.418 | 0.479 | 0.057 |
| DK | 9 | 105 | 0 | 2.46 | 2.733 | 0.455 | 0.523 | 0.074** |
| ShQ | 12 | 113 | 1 | 2.34 | 2.662 | 0.406 | 0.468 | 0.053** |
| LP | 20 | 142 | 1 | 2.83 | 2.961 | 0.432 | 0.523 | 0.140** |
| GD | 8 | 82 | 3 | 1.91 | 2.218 | 0.391 | 0.385 | -0.083 |
| YP | 21 | 144 | 2 | 2.69 | 2.969 | 0.457 | 0.536 | 0.182** |
| QY | 15 | 129 | 1 | 2.64 | 2.890 | 0.542 | 0.541 | -0.032 |

Notes: *A*_T_: the total number of alleles, *A*p: the number of private alleles, *A*e: the effective number of alleles, *R*s: the allele richness (standardized for four individuals using rarefaction), *H*o: the observed heterozygosity, U*H*e: unbiased expected heterozygosity, *F*_IS_: the inbreeding coefficient.
