# Supplementary material for: Molecular phylogeography and species distribution modelling evidence of ‘oceanic’ adaptation for Actinidia eriantha with a refugium along the oceanic–continental gradient in a biodiversity hotspot
Source: BMC Plant Biol. 2022 Feb 28;22:89. doi: 10.1186/s12870-022-03464-5 (PMC8883688; doi:10.1186/s12870-022-03464-5)
Supplement: Supplementary file 6 — Additional file 6. The most probable number of clusters determined using the Delta K approach. (a) When K = 2 for all individuals, Delta K has the highest value. (b) When K = 4 for western subset (plus HA and NJ) of individuals, Delta K has the highest value. [file 12870_2022_3464_MOESM6_ESM.pdf]

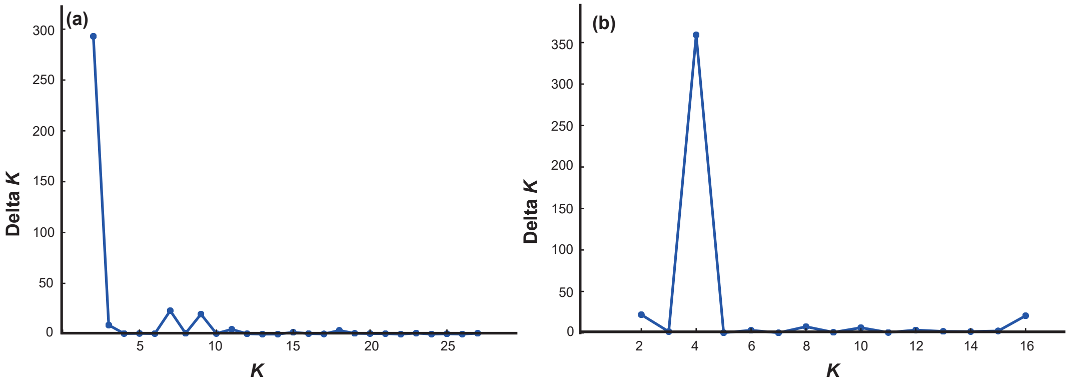

Additional file 6. The most probable number of clusters determined using the Delta *K* approach. (a) When *K* = 2 for all individuals, Delta *K* has the highest value. (b) When *K* = 4 for western subset (plus HA and NJ) of individuals, Delta *K* has the highest value.
