# Supplementary material for: Molecular phylogeography and species distribution modelling evidence of ‘oceanic’ adaptation for Actinidia eriantha with a refugium along the oceanic–continental gradient in a biodiversity hotspot
Source: BMC Plant Biol. 2022 Feb 28;22:89. doi: 10.1186/s12870-022-03464-5 (PMC8883688; doi:10.1186/s12870-022-03464-5)
Supplement: Supplementary file 7 — Additional file 7. Migration rates (m) across the five clusters of Actinidia eriantha based on 31 neutral nuclear SSRs. [file 12870_2022_3464_MOESM7_ESM.docx]

| Additional file 7 Migration rates (*m*) across the five clusters of *Actinidia eriantha* based on 31 neutral nuclear SSRs. | | | | | |
| --- | --- | --- | --- | --- | --- |
|  | Cluster I | Cluster II | Cluster III | Cluster IV | Cluster V |
| Mean historical migration rates | | | | | |
| Cluster I |  | 0.035 (0.024, 0.045) | 0.012 (0.005, 0.019) | 0.022 (0.016, 0.027) | **0.082** (0.089, 0.103) |
| Cluster II | 0.010 (0.005, 0.015) |  | 0.023 (0.016, 0.030) | 0.016 (0.011, 0.021) | 0.015 (0.004, 0.018) |
| Cluster III | 0.012 (0.005, 0.018) | 0.001 (0, 0.006) |  | 0.015 (0.009, 0.021) | 0.014 (0.008, 0.019) |
| Cluster IV | 0.031 (0.028, 0.040) | 0.034 (0.021, 0.041) | 0.019 (0.013, 0.024) |  | **0.169** (0.155, 0.173) |
| Cluster V | 0.022 (0.016, 0.027) | 0.016 (0.011, 0.022) | 0.007 (0.002, 0.012) | 0.045 (0.040, 0.051) |  |
| Mean recent migration rates | | | | | |
| Cluster I |  | 0.004 (0, 0.012) | 0.004 (0, 0.012) | 0.004 (0, 0.013) | 0.005 (0, 0.014) |
| Cluster II | 0.005 (0, 0.015) |  | 0.005 (0, 0.014) | 0.009 (0, 0.022) | 0.005 (0, 0.014) |
| Cluster III | 0.010 (0, 0.028) | 0.010 (0, 0.029) |  | 0.047 (0.012, 0.085) | 0.010 (0, 0.029) |
| Cluster IV | 0.003 (0, 0.008) | 0.003 (0, 0.008) | 0.003 (0, 0.008) |  | 0.006 (0, 0.014) |
| Cluster V | 0.001 (0, 0.003) | 0.001 (0, 0.003) | 0.001 (0, 0.003) | 0.001 (0, 0.004) |  |
| The source populations for migration are given in rows and populations receiving migrants are in columns. Migration rates > 0.05 are in bold. *m*_h_ = *Mμ* (*M* is the mutation–scaled effective immigration rate, *μ* is the mutation rate per site: 3 × 10^–4^). | | | | | |
