# Supplementary material for: Molecular phylogeography and species distribution modelling evidence of ‘oceanic’ adaptation for Actinidia eriantha with a refugium along the oceanic–continental gradient in a biodiversity hotspot
Source: BMC Plant Biol. 2022 Feb 28;22:89. doi: 10.1186/s12870-022-03464-5 (PMC8883688; doi:10.1186/s12870-022-03464-5)
Supplement: Supplementary file 9 — Additional file 9. Set A, B, C and D of scenarios in DIYABC of Actinidia eriantha. Posterior probability of each scenario obtained by logistic regression of 1% of the closest simulated datasets is shown on the top of the scenario. Scenario outlined in red is the best option. [file 12870_2022_3464_MOESM9_ESM.pdf]

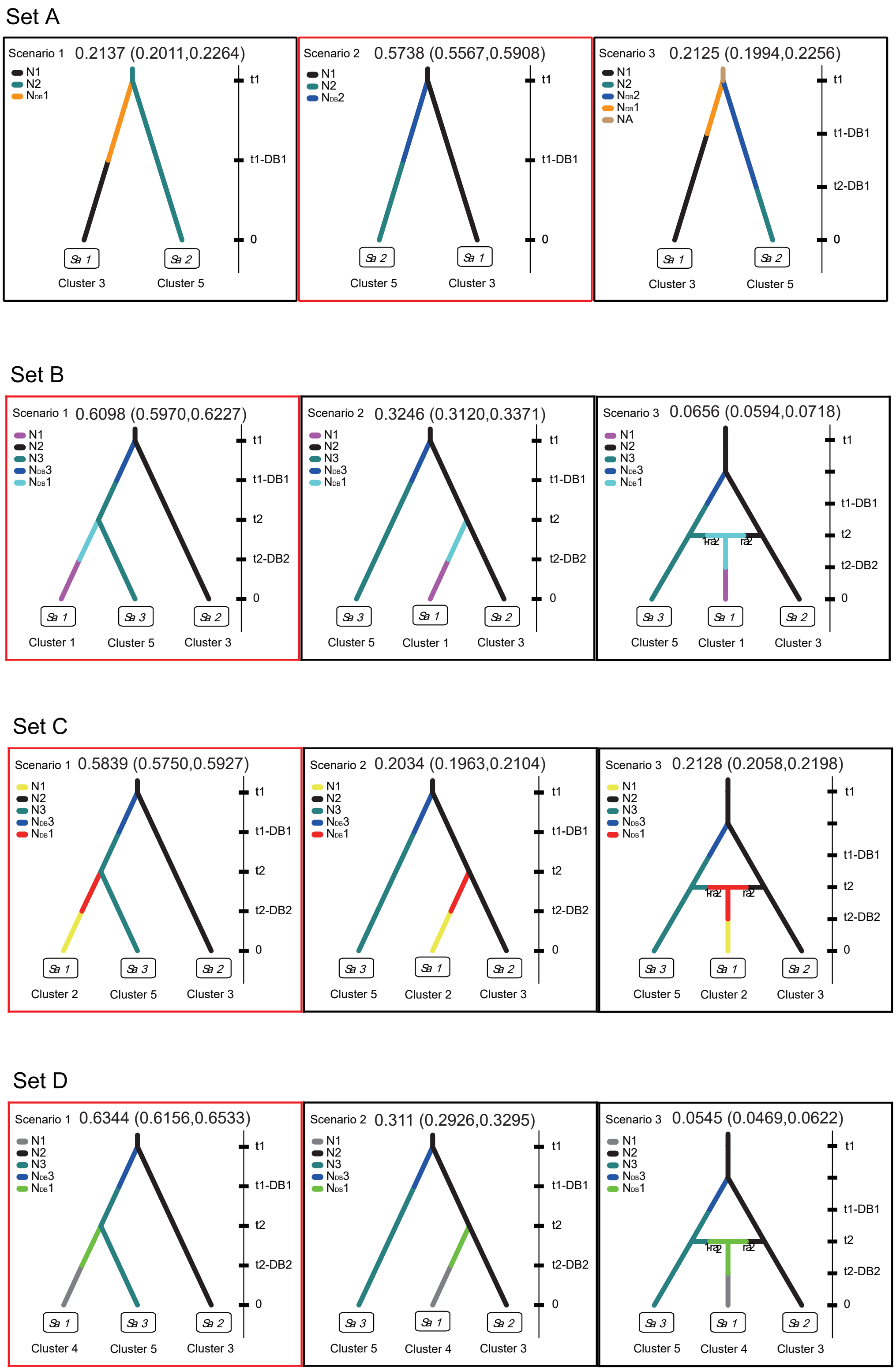

Additional file 9. Set A, B, C and D of scenarios in DIYABC of *Actinidia eriantha*. Posterior probability of each scenario obtained by logistic regression of 1% of the closest simulated datasets is shown on the top of the scenario. Scenario outlined in red is the best option.
