# Supplementary figures and images for: Molecular phylogeography and species distribution modelling evidence of ‘oceanic’ adaptation for Actinidia eriantha with a refugium along the oceanic–continental gradient in a biodiversity hotspot
Source: BMC Plant Biol. 2022 Feb 28;22:89. doi: 10.1186/s12870-022-03464-5 (PMC8883688; doi:10.1186/s12870-022-03464-5)

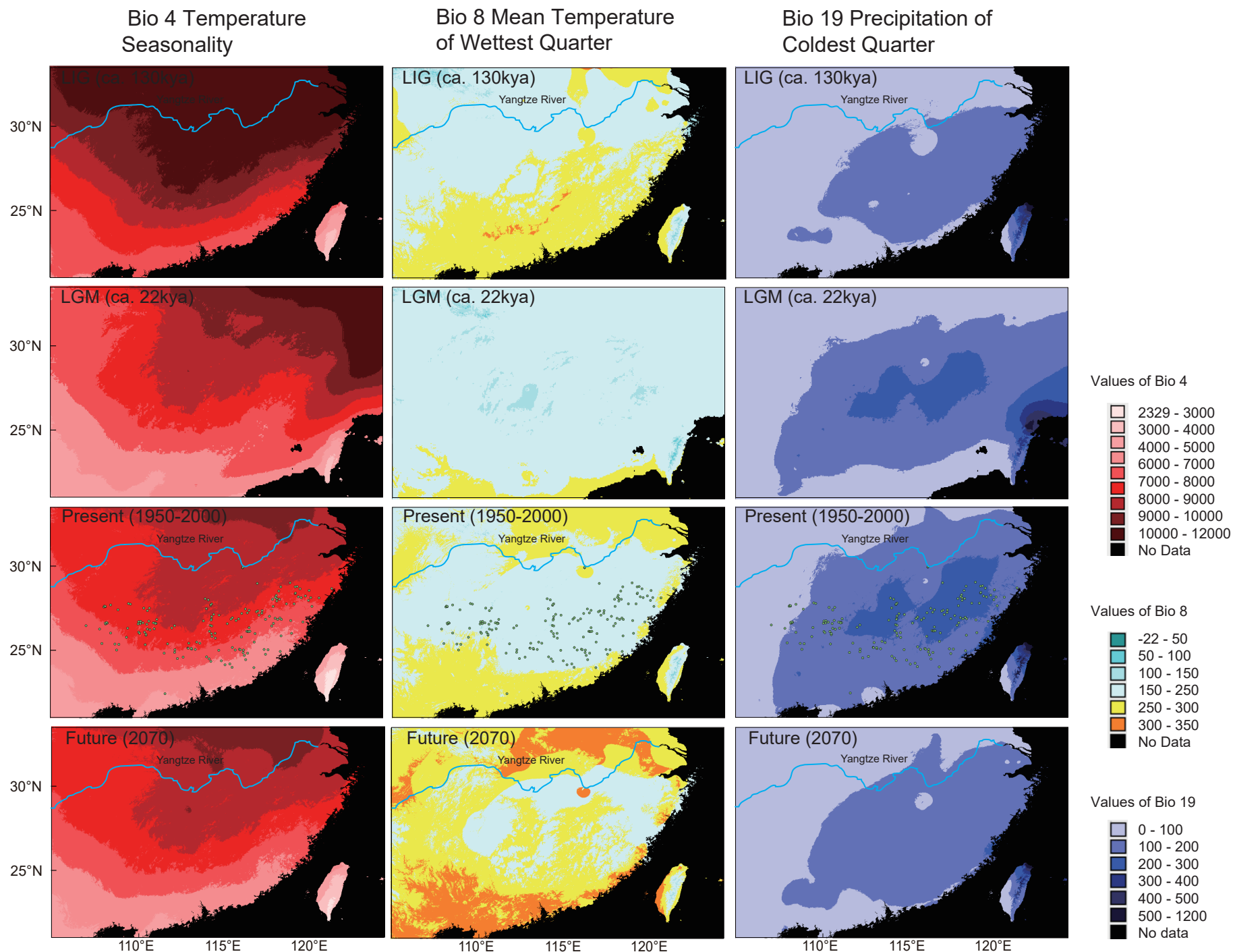

Additional file 11. The isolines in the three climate layers Bio 4, Bio 8 and Bio 19.

Supplement: Supplementary file 11 — Additional file 11. The isolines in the three climate layers Bio 4, Bio 8 and Bio 19. [file 12870_2022_3464_MOESM11_ESM.pdf]
