# Supplementary material for: Molecular phylogeography and species distribution modelling evidence of ‘oceanic’ adaptation for Actinidia eriantha with a refugium along the oceanic–continental gradient in a biodiversity hotspot
Source: BMC Plant Biol. 2022 Feb 28;22:89. doi: 10.1186/s12870-022-03464-5 (PMC8883688; doi:10.1186/s12870-022-03464-5)
Supplement: Supplementary file 13 — Additional file 13. The results of niche identity test between West group and East group in subtropical China. The histogram indicate the randomized distributions of Warren et al.’s I and Schoener’s D and the arrow indicates the observed values of I and D. The x-axis indicates values of I and D, and the y-axis indicates the number of randomizations. [file 12870_2022_3464_MOESM13_ESM.pdf]

Identity test

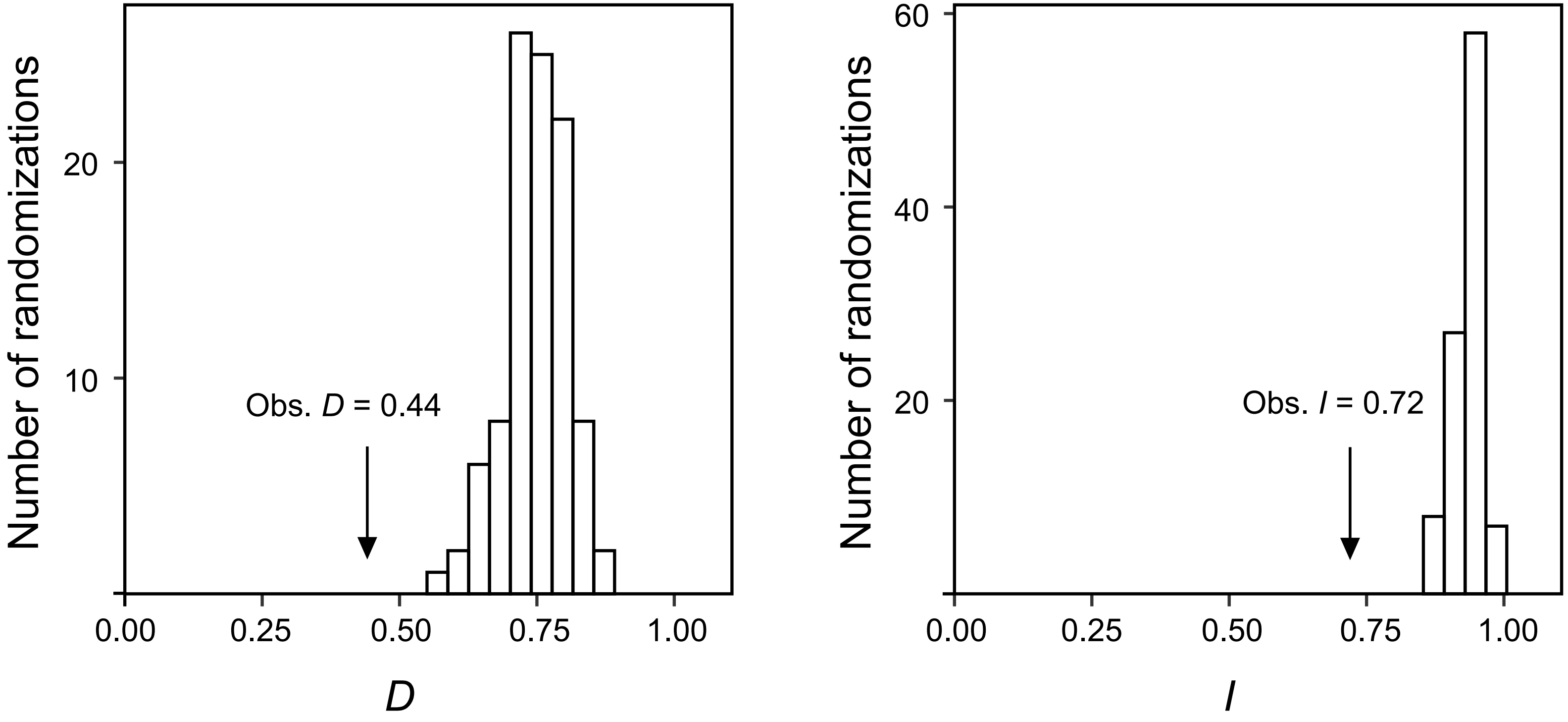

Additional file 13. The results of niche identity test between West group and East group in subtropical China. The histogram indicate the randomized distributions of Warren et al.'s *I* and Schoener’s *D* and the arrow indicates the observed values of *I* and *D*. The x-axis indicates values of *I* and *D*, and the y-axis indicates the number of randomizations.
