# Supplementary material for: Molecular phylogeography and species distribution modelling evidence of ‘oceanic’ adaptation for Actinidia eriantha with a refugium along the oceanic–continental gradient in a biodiversity hotspot
Source: BMC Plant Biol. 2022 Feb 28;22:89. doi: 10.1186/s12870-022-03464-5 (PMC8883688; doi:10.1186/s12870-022-03464-5)
Supplement: Supplementary file 14 — Additional file 14. cpDNA haplotype with its Gene Bank accession number. [file 12870_2022_3464_MOESM14_ESM.docx]

| Additional file 14 cpDNA haplotype with its Gene Bank accession number. | | | |
| --- | --- | --- | --- |
| Haplotype | *ndhF*-*rpl132* | *rps*16-*trn*Q | *trn*E-*trn*T |
| H1 | MN974334 | MN974358 | MN974310 |
| H2 | MN974335 | MN974359 | MN974311 |
| H3 | MN974343 | MN974367 | MN974319 |
| H4 | MN974344 | MN974368 | MN974320 |
| H5 | MN974345 | MN974369 | MN974321 |
| H6 | MN974341 | MN974365 | MN974317 |
| H7 | MN974342 | MN974366 | MN974318 |
| H8 | MN974337 | MN974361 | MN974313 |
| H9 | MN974338 | MN974362 | MN974314 |
| H10 | MN974339 | MN974363 | MN974315 |
| H11 | MN974340 | MN974364 | MN974316 |
| H12 | MN974328 | MN974352 | MN974304 |
| H13 | MN974329 | MN974353 | MN974305 |
| H14 | MN974330 | MN974354 | MN974306 |
| H15 | MN974346 | MN974370 | MN974322 |
| H16 | MN974333 | MN974357 | MN974309 |
| H17 | MN974325 | MN974349 | MN974301 |
| H18 | MN974336 | MN974360 | MN974312 |
| H19 | MN974332 | MN974356 | MN974308 |
| H20 | MN974331 | MN974355 | MN974307 |
| H21 | MN974326 | MN974350 | MN974302 |
| H22 | MN974327 | MN974351 | MN974303 |
| H23 | MN974324 | MN974348 | MN974300 |
| *Actinidia chinensis* | MN974323 | MN974347 | MN974299 |
| *A. fulvicoma* 1 | MW387125 | MW387124 | MW387126 |
| *A. fulvicoma* 2 | MW387122 | MW387121 | MW387123 |
| *A. fulvicoma* 3 | MW387119 | MW387118 | MW387120 |
